# Detecting hybridization in Chilean species of the genus *Baccharis* L.

**DOI:** 10.1111/plb.13751

**Published:** 2024-12-09

**Authors:** F. Schneider, F. Hellwig

**Affiliations:** ^1^ Systematic Botany with Herbarium Haussknecht and Botanical Garden, Institute of Ecology and Evolution, Faculty of Biological Sciences Friedrich‐Schiller‐Universität Jena Germany

**Keywords:** *Baccharis linearis*, *Baccharis macraei*, *Baccharis* × *intermedia*, GBS, homoploid hybrid, hybrid swarm, population genetics

## Abstract

- The genus *Baccharis* in Chile is an extraordinary example of admixture, previously described only morphologically and chemically. In Chile, the genus forms a homoploid complex with at least 16 species and 21 hybrids.
- Genotyping‐by‐Sequencing (GBS) was used to clarify the hybrid character of *Baccharis* × *intermedia*, which originated from the species *B. macraei* and *B. linearis*. Additionally, *B. vernalis*, another species with a morphological resemblance to *B. macraei*, was subjected to analysis to ascertain its role in the hybridization process.
- A total of 11,006 SNPs and 72 individuals were analysed using Treemix, *D*‐ and *f*‐statistics, which revealed genetic evidence of hybridization between *B. macraei* and *B. linearis*. Furthermore, other genetic indicators, such as a high level of heterozygosity, also provided evidence of the hybrid nature of *Baccharis* × *intermedia*. Additionally, one individual exhibited strong genetic proportions derived from *B. vernalis*, *B. macraei*, and *B. linearis*. Distinct individuals were clustered using sparse Non‐Negative Matrix Factorization (sNMF) into five distinct groups, representing the described species and the hybrid. *B. macraei* exhibited division into a northern and a southern subpopulation.
- The morphological and chemical evidence of the hybrid character of *Baccharis* × *intermedia* is corroborated by genetic data. Further, the most likely evolutionary scenario is a hybrid swarm. Genetic differentiation between *B. linearis* and *B. macraei* indicates separation prior to secondary contact. The close relationship of *B. macraei* and *B. vernalis* was confirmed, suggesting that it may emerge as a vicariant species on different soil types.

## Graphical abstract

Genotyping‐by‐sequencing was used to clarify the hybrid character of *Baccharis* × *intermedia*, originating from the species *B. macraei* and *B. linearis*.

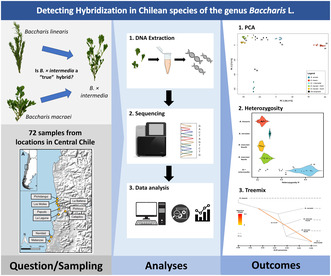

## INTRODUCTION

*Baccharis* L. (Asteraceae, Astereae, Baccharidinae) is one of the largest genera within the tribe Astereae, with about 354 (Müller 2013) to 500 (Malagarriga‐Heras 1977) species. In Chile 16 species occur (Hellwig 1990). Furthermore, 21 hybrids of the above‐mentioned 16 parental species have been described based on morphology in the same geographic area. *Baccharis* is a genus that is native to the majority of Chilean territory. However, it is absent between 18° and 28°30′ S. This region is characterized by an almost vegetation‐free desert, as well as the so‐called “Loma” vegetation on coasts. Furthermore, no species can be found in the “travesía” (a dwarf shrub heath) between Copiapó and Vallenar (Hellwig 1990). Hybridization occurs when two or more species coexist in sympatry (Hellwig 1990). All hybrids and their parental species described so far using chromosome counts are homoploid (Hellwig 1990). Rapid hybridization between lineages is also observed in young plant taxa within other genera (e.g., *Sphagnum* and *Citrus*: Olena *et al*. 2018; Wu *et al*. 2021). Weak genetic differentiation in such young species may make them prone to hybridize at the homoploid level.

The center of diversity for *Baccharis* lies in the Andes and south‐eastern Brazil (Luis 1955). Previous studies on Chilean *Baccharis* species have mostly focused on morphological characteristics for delimitation of the genus, its species, and hybrids. Species can be distinguished using leaf, floral head and indument characteristics (Hellwig 1990, 1992). From the morphological data available, it is assumed that *Baccharis* in Chile forms a complex hybrid swarm (compare Schumer *et al*. 2014 for the concept) or syngameon *sensu* Grant (1976).

Currently no genetic studies have been conducted researching homoploid reticulate evolution in Chilean *Baccharis*. This study focused on *B. macraei*, *B. linearis*, and their putative hybrid *B. × intermedia*. *Baccharis × intermedia* was first described by Candolle & Candolle (1836, p. 411) as *Baccharis intermedia*, with intermediate characteristics between *B. rosmarinifolia* and *B. concava*: “Media feré inter *B. rosmarinifoliam* et *concavam*. (v.s.)”. *Baccharis rosmarinifolia* is a synonym for *B. linearis*, and the description of Candolle & Candolle (1836) for *B. concava* fits with the current species description of *B. vernalis* (see Hellwig 1990 for nomenclatural details). *Baccharis vernalis* is vegetatively very similar to *B. macraei*, a species not described by DeCandolle, and can only be distinguished by its habitat, flowering period, and flower morphology. *Baccharis macraei* appears on multiple coastal slopes extending beyond dunes in arid open habitats on sandy soil. In contrast, *B. vernalis* is documented to thrive on loamy, stony slopes along the coastline (see Hellwig 1990). Later, Hellwig (1990) identified *B. × intermedia* as a hybrid between *B. macraei* and *B. linearis*. Yet due to the similarity of *B. macraei* and *B. vernalis*, it cannot be excluded that both species are involved in the formation of the hybrid *B. × intermedia*. *Baccharis linearis* occurs in Central Chile, but only reaches the coast in few localities, while *B. macraei* only grows on sandy soil of the coastal dunes. *Baccharis × intermedia* occurs only where both species are sympatric. *Baccharis linearis* and *B. macraei* overlap in their flowering period in February and March (Hellwig 1990).

In a study by Faini *et al*. (1991), the putative parental species *B. macraei* Hook. & Arn., *B. linearis* (Ruiz & Pav.) Pers. of *B. × intermedia* DC. was examined chemically and morphologically. Morphologically, the hybrid shows intermediate leaf shape, capitulum arrangement, and branch angles. Chemical compounds (mainly terpenes and flavonoids) in *B. × intermedia* showed additive inheritance from both parental species. The authors concluded that *B. × intermedia* consists of a swarm of hybrids between the parental species *B. linearis* and *B. macraei*. Chromosome counts show that the hybrid nothospecies *B. × intermedia*, as well as its parental species, are diploid (Hellwig 1990, and unpublished data from F. Hellwig).

Recent advances in genomic analysis allow testing of the hybrid nature of *Baccharis × intermedia* using next generation sequencing. Samples from each of these species were analysed using population genetic methods, representing the first assessment based on molecular evidence of a homoploid hybrid complex in Chilean *Baccharis*. Our study asked: (i) to what degree are *B. linearis* and *B. macraei* genetically separated; (ii) is *B*. × *intermedia* a hybrid resulting from gene flow between *B. linearis* and *B. macraei* after secondary contact; and (iii) can the patterns of genetic variation be explained including the two parental species alone or are there indications of admixture from another sympatric *Baccharis* species (e.g., *B. vernalis*)?

## MATERIAL AND METHODS

### Sample collection

**Fig. 1:**
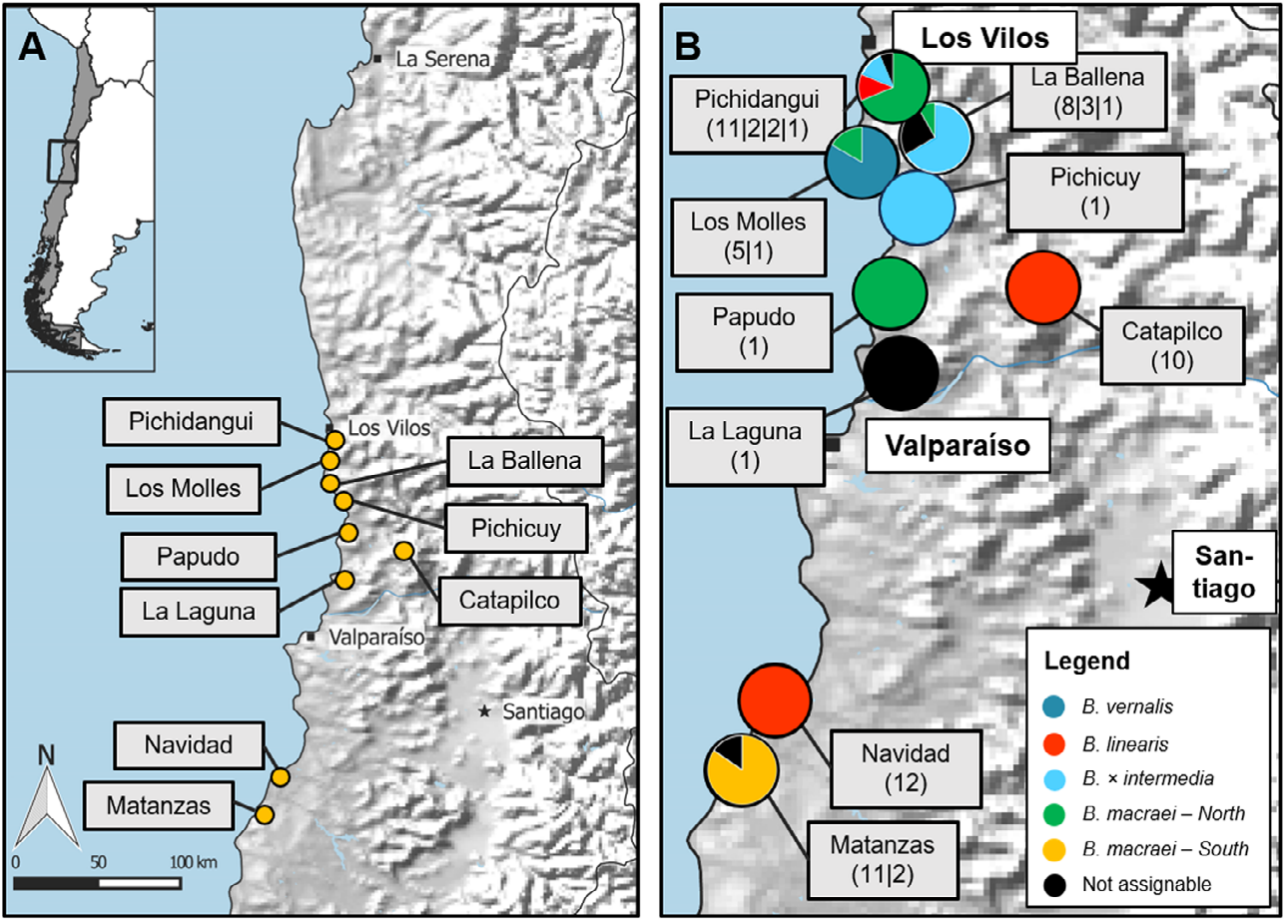
(A) Locations in Chile where *Baccharis* spp. were sampled. (B) Taxon diversity per location resulting from the sNMF clustering. Numbers indicate number of individuals per location and per cluster (for *K* = 5; individuals labelled as not assignable have a minimum of 25% admixture from another cluster).

In March 2019, 72 plant tissue samples and herbarium vouchers were collected from multiple locations in Chile (Fig. 1). The tissue samples were stored in 96% ethanol and later transferred to Germany for further analyses. The collected individuals putatively belong to the species: *Baccharis linearis*, *B. macraei*, and their corresponding hybrid *B. × intermedia* (Hellwig 1990). Also, some individuals of *B. vernalis* were sampled. These samples were collected from nine different locations in the coastal region of Central Chile, around Valparaíso (Fig. 1). The species were determined in the field using information provided in Hellwig (1990). The collected individuals per species and their respective location in Chile are given in Table 1. Matanzas can be considered a reference population for *B. macraei*, and Navidad and Catapilco for *B. linearis*. Clear hybrid localities where species appear sympatrically are Pichidangui and La Ballena. The voucher specimens were dried, shipped to Germany, and deposited in the Herbarium Haussknecht (JE).

**Table 1:** Collected individuals per species and their respective location in Chile, with their respective number of samples.

taxa	no. of samples	location
*Baccharis macraei*	26	Matanzas (12) Pichidangui (11) Papudo (1) La Laguna (1) Los Molles (1)
*Baccharis linearis*	22	Navidad (11) Catapilco (10) Pichidangui (1)
*Baccharis × intermedia*	18	La Ballena (12) Pichidangui (4) Pichicuy (1) Navidad (1)
*Baccharis vernalis*	6	Los Molles (5) Matanzas (1)

### DNA extraction and sequencing

Tissue samples were shipped from Chile in 96% ethanol. The samples were stored in ethanol for subsequent chemical analyses, which are not pertinent to this study. The samples were kept in ethanol for approximately 2 months. Some of the samples also had dried replicas. No differences were identified in the genetic analyses between the dried samples and those stored in ethanol. It was therefore decided to utilize the complete set of samples stored in ethanol. Extraction, sequencing and genotyping by sequencing (GBS) was performed by LGC Genomics (Berlin, Germany; https://www.lgcgroup.com/). DNA was isolated with the sbeadexTM mini plant kit from LGC Genomics. Lysis buffer was additionally mixed with 1% thioglycerol and RNase. All DNA extracts were eluted in Tris buffer containing EDTA (10 mM Tris, 0.1 mM EDTA).

Genotyping by sequencing (GBS) was used to generate genome‐wide polymorphism data (Elshire *et al*. 2011). The libraries for GBS were prepared using the enzyme combination PstI‐ApeKI, and pooling was done for 2 × 150 bp sequences on an Illumina NextSeq 500. For library preparation, the LGC—Library preparation protocol (see Appendix S7) was used. Sequencing aimed for an average 1.5 million reads per sample. Insert size mean range was approximately 180 bp.

### Raw read processing

For the whole dataset a total of 368,723,466 raw reads where gathered. The reads were demultiplexed sample‐wise using the Illumina bcl2fastq v. 2.17.1.14 software. For further processing, filters were applied using Illumina bcl2fastq v. 2.17.1.14 as described below. (1) One or two mismatches or Ns were allowed in the barcode read when the barcode distances between all libraries on the lane allowed it; also, no mismatches or Ns were allowed in the inline barcodes, but Ns were allowed in the restriction site. (2) Clipping of sequencing adapter remnants from all reads. (3) Reads with final length < 20 bases were discarded. (4) Restriction enzyme site filtering at 5′ ends of reads. (5) Removal of all reads containing Ns. (6) Trimming of reads at 3′‐end to obtain a minimum average Phred quality score of 20 over a window of 10 bases. After these steps, the combined reads were clustered with CD‐HIT‐EST, allowing up to 5% difference, and singletons were excluded (Huang *et al*. 2010).

In the absence of a whole *Baccharis* genome, the reference sequence was generated from the overall sequence of every individual. The average nucleotide was set as the reference nucleotide at a specific position. For alignment and creation of the reference, the software Bowtie2 v. 2.2.3 was used.

The variant discovery and genotyping of samples was done with the software Freebayes v. 1.0.2–16 (Garrison & Marth 2012). The following specific parameters were used: minimum base quality = 10, minimum supporting allele qsum = 10, read mismatch limit = 3, minimum coverage = 5, no indels, minimum alternate count = 4, exclude unobserved genotypes, ploidy = 2, no multi‐nucleotide polymorphisms (mnps), no‐complex, mismatch base quality threshold = 10. The locus count for all samples was 63,348, with a mapping rate from 93%. In total, 4,429 total polymorphic loci were counted, with a total number of 87,606 SNPs across all samples. Following this procedure, the variants were filtered using a GBS‐specific rule set using Freebayes v. 1.0.2–16: (1) the read count for a locus must exceed eight reads; (2) genotypes must have been observed in at least 64% of samples; (3) minimum allele frequency across all samples must exceed 5%. After this procedure, the outcome was 11,007 SNPs, which were further used for population genetics analyses. One SNP had to be excluded from further analysis as it was invariant (see Table 2). The data were stored in the variant call format v. 4.1 (vcf; Danecek *et al*. 2011). Subsequently, population genetics analyses were conducted anew, after filtering for linkage disequilibrium (for details and results, see Appendix S8). Following application of the linkage disequilibrium filter, no significant discrepancies between the results were identified.

**Table 2:** Results of the GBS analysis comprising all samples of *Baccharis linearis*, *B. macraei*, *B. vernalis*, and *B. × intermedia*.

Cluster locus count	63,348
Mapping rate	93%
Polymorphic locus count	4,429
Total number of SNPs across all samples	87,606
Total number of SNPs across all samples with minimum read count of 8	74,083
Total number of fully covered SNPs in 66% of samples, with allele frequency at or above 5% and minimum read count of 8	11,006
Total number of fully covered SNPs in 66% of samples, with allele frequency at or above 10% and minimum read count of 8	7,570

### Principal components analysis (PCA)

For the PCA (Pearson 1901; Hotelling 1933) the .vcf‐file was converted to a .geno‐file and an .indv‐file using pdg‐spider v. 2.1.1.5 (Lischer & Excoffier 2012). PCA and plotting were done in R v.3.6.0, using the LEA package v. 2.6.0 for the PCA (Frichot & François 2015; R Core Team 2019), and the package ggplot2 v. 3.3.3 (Wickham 2016) for plotting. Default analysis settings were used for PCA. All 72 samples were used in the PCA. Plotting was performed twice: first with the field labels of samples, and second with groups derived from clustering. Those samples that combined features from several groups and with <75% of the predominant group were not assigned to a specific group a priori, but rather are represented as black dots and are labelled “unassignable” in Fig. 3.

### Sparse non‐negative matrix factorization (sNMF)—Clustering

To further explore the genetic substructure of the dataset (72 individuals, 11.006 SNPs), samples were clustered via a non‐spatial, non‐Bayesian approach provided in the LEA package v. 2.6.0, in R v. 3.6.0.

The sNMF has least squares optimization and clusters faster than Bayesian methods such as “structure” or “TESS 2.3” (Frichot *et al*. 2014). The sNMF is model free and makes no assumptions regarding the underlying biological processes. It is robust to departures from population genetic models, in contrast to “structure”, which uses Hardy–Weinberg models (Pritchard *et al*. 2000).

Clustering was performed with different numbers of clusters (1–20), a regularization parameter *α* = 5, tolerance parameter *ε* = 10^−4^, a 5% of masked genotypes when computing the cross‐entropy criterion, and 100 repetitions per cluster. Model fit and estimation of the number of clusters is based on the cross‐entropy value (Wold 1978; Eastment & Krzanowski 1982; Frichot *et al*. 2014). “CLUMPAK” was used (Kopelman *et al*. 2015) to summarize the different runs. The best run of the chosen clusters was visualized with the packages pophelper v. 2.3.1 (Francis 2017), dplyr v. 1.0.2 (Wickham *et al*. 2020), and ggplot2 v. 3.3.3. Using the morphology of the individuals, the clusters could then be assigned to a specific taxon using the Hellwig (1990) species description.

### Hierarchical cluster Analysis and neighbour‐joining tree

To obtain an overview on the genetic structure of the data, a Hierarchical Cluster Analysis was performed, and a Neighbour‐Joining Tree built with R v. 3.6.0. A pairwise genetic distance matrix was created for all individuals with the “dist.gene” function in ape v. 5.4.1 of Paradis & Schliep (2019). These algorithms were applied to find a suitable outgroup in the dataset and visualize the degree of genetic isolation between the different species. Outgroup identification is further needed to distinguish divergent evolution between closely related species and introgression. For this purpose, we applied Treemix (Pickrell & Pritchard 2012), see below, an algorithm that requires inclusion of an outgroup.

For the Hierarchical Cluster Analysis, a distance matrix was created and then analysed with the “hclust” command. “Ward.D2” was chosen as criterion for clustering, representing Ward Jr's (1963) clustering (Murtagh & Legendre 2014). The unrooted Neighbour‐Joining Tree was calculated with the R‐package ape v. 5.4.1. (Saitou & Nei 1987).

### Treemix

To construct a tree topology including potential hybridization events, Treemix v. 1.13 was used (Pickrell & Pritchard 2012). The analysis was performed ranging from zero to 11 migration events, while using 500 bootstrap repeats with sample size correction. The maximum likelihood was plotted to estimate the amount of migration events. The same outgroup (two individuals from *B. linearis*) as for *D*‐statistics and *f*‐statistics was chosen. The Treemix – tree and the residuals were plotted in R v. 3.6.0.

### Heterozygosity analysis

Percentage of heterozygous alleles was measured over all SNPs, excluding missing data. Individuals were assigned to a specific taxon based on their cluster results. For each taxon frequency, distribution of that percentage was presented as a violin plot (Fig. 5) using the package ggplot2 v. 3.3.3. Percentage of heterozygous alleles for every individual was then correlated to percentage of the “*B. × intermedia* cluster” in the individual genomes resulting from the sNMF‐clustering. The “Pearson” method was used for correlation. Visualization was done in ggplot2 v. 3.3.3, while using a generalized linear model for the regression.

### *F*_ST_ , *F*‐statistic, and *D*‐statistic

Additionally, to identify a suitable outgroup and to elucidate relationships among the groups that resulted from clustering, both WC‐F_ST_ and Nei‐F_ST_ were performed. *F*_ST_‐values were estimated via pairwise WC‐F_ST_, including only genetic drift as factor (Weir & Cockerham 1984), and pairwise Nei‐F_ST_, considering mutation and genetic drift (Nei 1987). Both methods were applied as implemented in the package “hierfstat” v. 0.04‐22 in R v. 3.6.0. (Goudet & Jombart 2021).

To describe how populations are related to each other, *f*_2_, *f*_3_, and *f*_4_ statistics were used. *f*_2_ measures the amount of genetic drift – frequency change of an allele along a graph edge that separates two populations. *f*_2_ statistics were calculated with Admixtools v. 7.0.2 (Patterson *et al*. 2012). To test whether a population is admixed, *f*_3_ and *f*_4_ were calculated. Negative *f*_3_ values correspond to a branch with negative genetic drift, which is not allowed under the null assumption and is an indicator for hybridization (Peter 2016). *f*_4_ measures the amount of drift that is shared between two population pairs. It is the covariance of allele frequency differences in each pair. *f*_3_ and *f*_4_ partition genetic drift into a component that is specific to single populations and a component that is shared between a pair of populations. By measuring only the *shared* drift between pairs of populations, statements about their evolutionary relationships can be made (Patterson *et al*. 2012; Peter 2016). *f*_3_ and *f*_4_ statistics were calculated with Treemix v. 1.13. In order to detect and measure hybridization; Patterson's *D* statistic was calculated with Dsuite v. 0.4 using Jackknife = 2,000 (Malinsky *et al*. 2021). Dsuite also calculated the *f*_4_ ratio, BBAA, ABBA, BABA, and *f*_3_. For calculating *f*_3_, the data were divided into seven blocks of 500 bp each. Based on results of the Hierarchical Cluster Analysis, Neighbour‐Joining Tree, and *F*_ST_ analysis, two individuals identified as *B. linearis* (number 25 and 35) were selected as the outgroup.

## RESULTS

### Genetic relationships in *Baccharis* populations

**Fig. 2:**
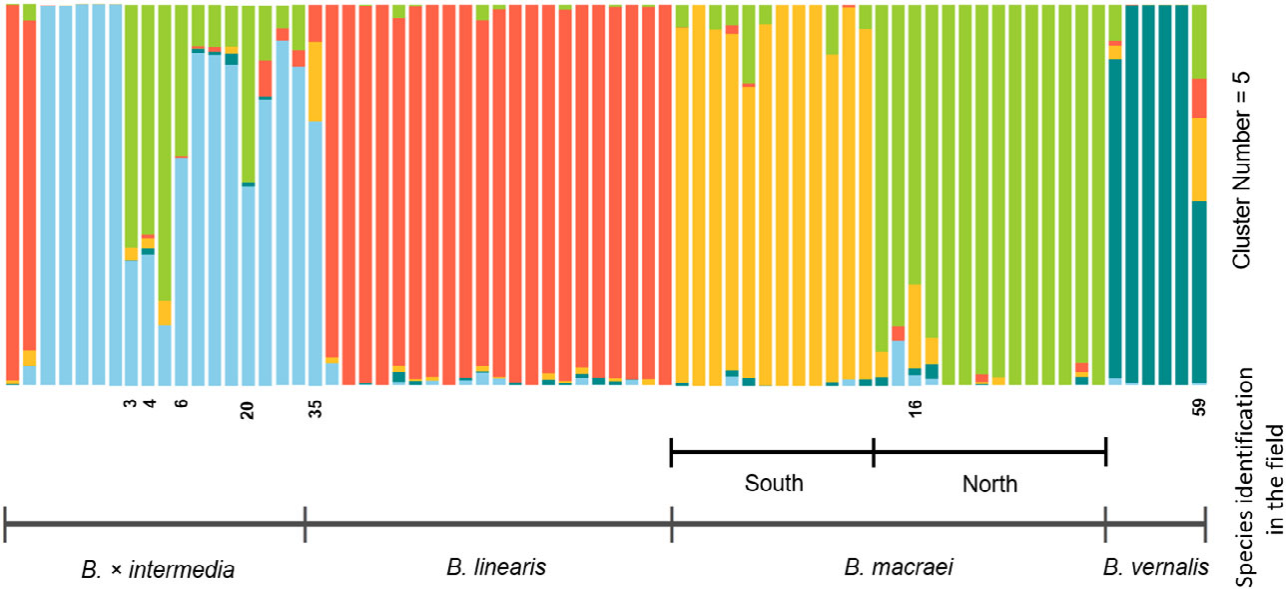
Genetic cluster assignment of the investigated four *Baccharis* taxa in Chile using LEA v. 2.6.0, shown as barplot. Vertical bars denote individuals. Five clusters were retained, indicated by different colours. Samples are ordered by preliminary field determination. *Baccharis macraei* separates into two clusters corresponding to their geographic distribution in a latitudinal gradient.

**Fig. 3:**
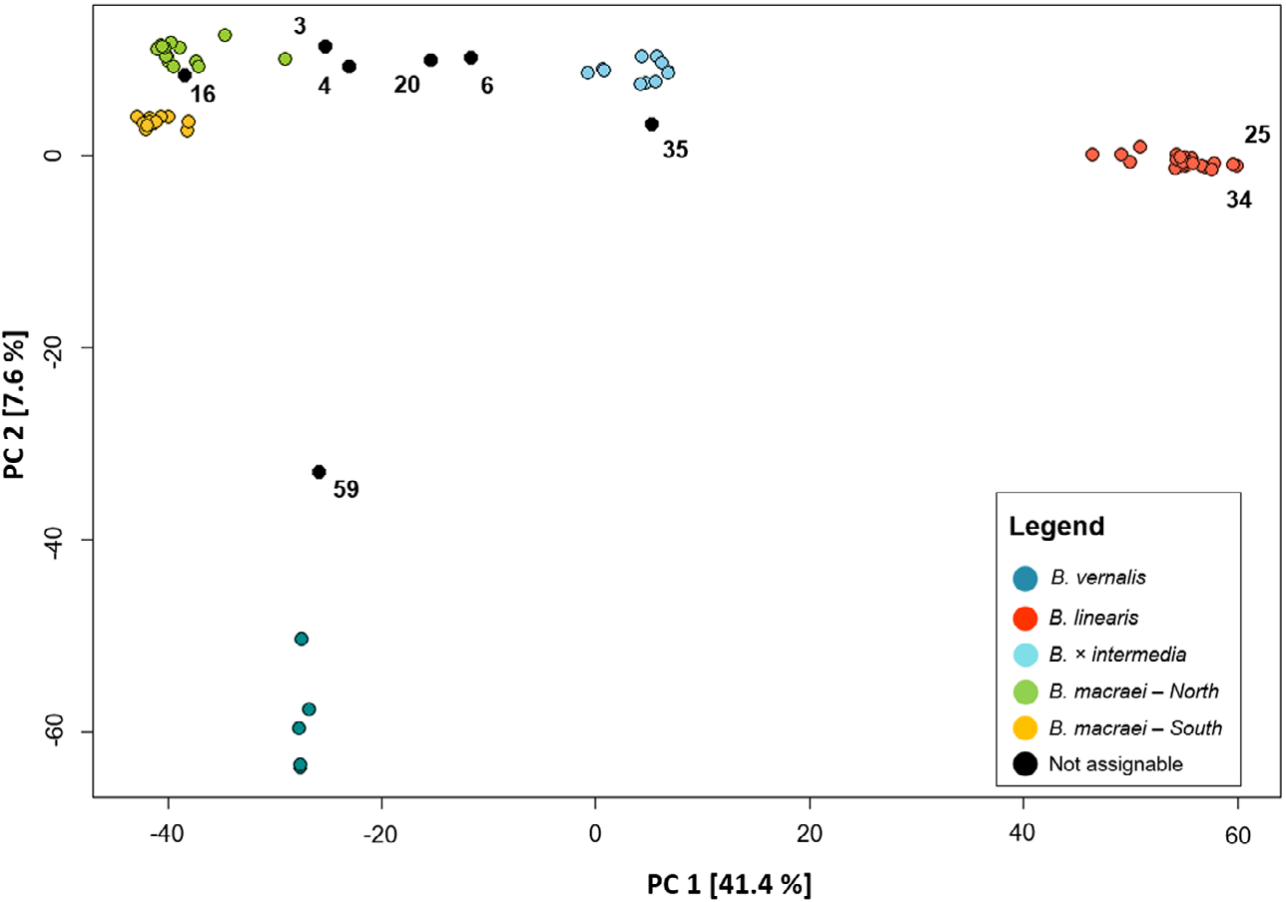
PCA results for four taxa of *Baccharis* L. in Chile. Shown are axis PC 1 (41.4%) and PC 2 (7.6%). Assignment of individuals is done according to the sNMF using *K* = 5 clusters. *Baccharis macraei* was split into a northern and southern cluster. Grouping into the category “Not assignable” was done for individuals that belong >25% to a different group in the sNMF clustering. Numbers represent individual ID.

The sampling of populations across the coastal region of Central Chile (ca. 300 km) provides an overview of their genetic structure. For data analysis, a clustering was first performed. Mean cross entropy level and Occam's razor led to selection of five clusters (Appendix S1; Fig. 2). This figure contains information for each individual and their putative species assignment based on morphological features in the field. Most of the samples were ascribed to their corresponding *a priori* determination. Within *Baccharis macraei*, two subgroups were distinguished. Considering the locations of the samples, a northern group of populations is separated from a southern group. Samples assigned to *Baccharis × intermedia* form a group well separated from the other species. Individuals between the northern *B. macraei* cluster and *B. × intermedia* were considered as not belonging to one of the groups (number 3, 4, 6 and 20) and thus were labelled “Not assignable” (Figs. 2 and 3). Two individuals assigned to *B. × intermedia* via *ad‐hoc* field determination were found to belong to the *B. linearis* cluster. A subsequent examination of their morphology revealed no evidence of intermediate features, indicating that the initial misidentification occurred in the field.

Plants clearly belonging to the *B. × intermedia* cluster also contain some portion found to be predominant in the northern *B. macraei* cluster. On the other hand, both putative parental species of *B. × intermedia* have some proportion of the *B. × intermedia* cluster. Sample number 59 was identified in the field as *B. vernali*s but in the clustering. almost half falls into other groups, i.e., *B. linearis* and *B. macraei*. Also, one individual in the *B. × intermedia* cluster (number 35) has a clear relationship to the southern *B. macraei* and *B. linearis*, while all other samples in *B. × intermedia* contain proportions of *B. linearis* and the northern subgroup of *B. macraei*. Alternatively, using two clusters in this analysis (see Appendix S1), shows a clear distinction between the groups *B. linearis* on one side and *B. macraei* and *B. vernalis* on the other. The putative hybrid *B. × intermedia* belongs in equal parts to the former mentioned clusters, indicating a hybrid origin.

A PCA was used to obtain an overall impression of the structure of the dataset. The different *Baccharis* species are well separated from each other, forming distinct groups (Fig. 3) including the putative hybrid *Baccharis × intermedia*, which is placed halfway between *B. macraei* and *B. linearis*. The hybrid is connected to *B. macraei* by a few individuals (number 3, 4, 6 and 20) that are not assigned to any taxon here. On the other hand, *B. × intermedia* is well separated from *B. linearis* without intermediate individuals. Individuals identified as *B. macraei* are grouped into two smaller clusters. The most informative principal components (PC) in the PCA are by far PC 1 and PC 2 (Appendix S2). Axis 1, with a PC of 41.4%, explained by far the most variation in the dataset. In comparison, Axis 2 only has a PC of 7.6%. For the following analysis, every non‐assignable individual was excluded.

### Genetic distance between the species

Nei's‐F_ST_ and WC‐F_ST_ were used to measure genetic distance between the different groups (Table 3). For Nei's distance, it is expected that the value is proportional to evolutionary time, when both the effects of mutation and genetic drift are considered (Nei 2013). Nei's‐F_ST_ and WC‐F_ST_ are quite similar, therefore WC‐F_ST_ results are only presented in Appendix S3. *F*_ST_ = 0 means no structure and no differentiation between groups, while *F*_ST_ = 1 means complete differentiation. In the dataset, *B. linearis* has the largest distance to all other groups, ranging from 0.155 to *B. vernalis* down to 0.092 to the putative hybrid *B. × intermedia. B. × intermedia* is equidistant to its putative parents, *B. linearis* and *B. macraei*. Within *B. macraei*, the northern populations are closer to the hybrid (Nei's‐F_ST_ 0.067) than the southern populations (Nei's‐F_ST_ 0.089). *B. vernalis* is closest to *B. macraei* (Nei's‐F_ST_: 0.086 and 0.093 to the northern and southern populations, respectively).

**Table 3:** Genetic distances (Nei's F_ST_) between species of *Baccharis* in Chile.

	*B. × intermedia*	*B. linearis*	*B. macraei—North*	*B. macraei—South*	*B. vernalis*
*B. × intermedia*	–				
*B. linearis*	0.089	–			
*B. macraei—North*	0.067	0.135	–		
*B. macraei—South*	0.089	0.144	0.039	–	
*B. vernalis*	0.118	0.154	0.086	0.093	–

In addition to determination of *F*_ST_ values, other distance‐based methods were also used. An unrooted Neighbour‐Joining Tree and a hierarchical clustering dendrogram were inferred (Appendix S4). Both methods yielded similar results: *B. linearis* is the most distant group to *B. macraei*, *B. vernalis*, and *B. × intermedia*. *Baccharis × intermedia* is placed between *B. linearis* and *B. macraei* and *B. vernalis*. In the hierarchical clustering, *B. vernalis* is an outgroup to *B. macraei* north and south. This is also reflected in the unrooted Neighbour‐Joining Tree.

### Relationships of *Baccharis* × *intermedia*

**Fig. 4:**
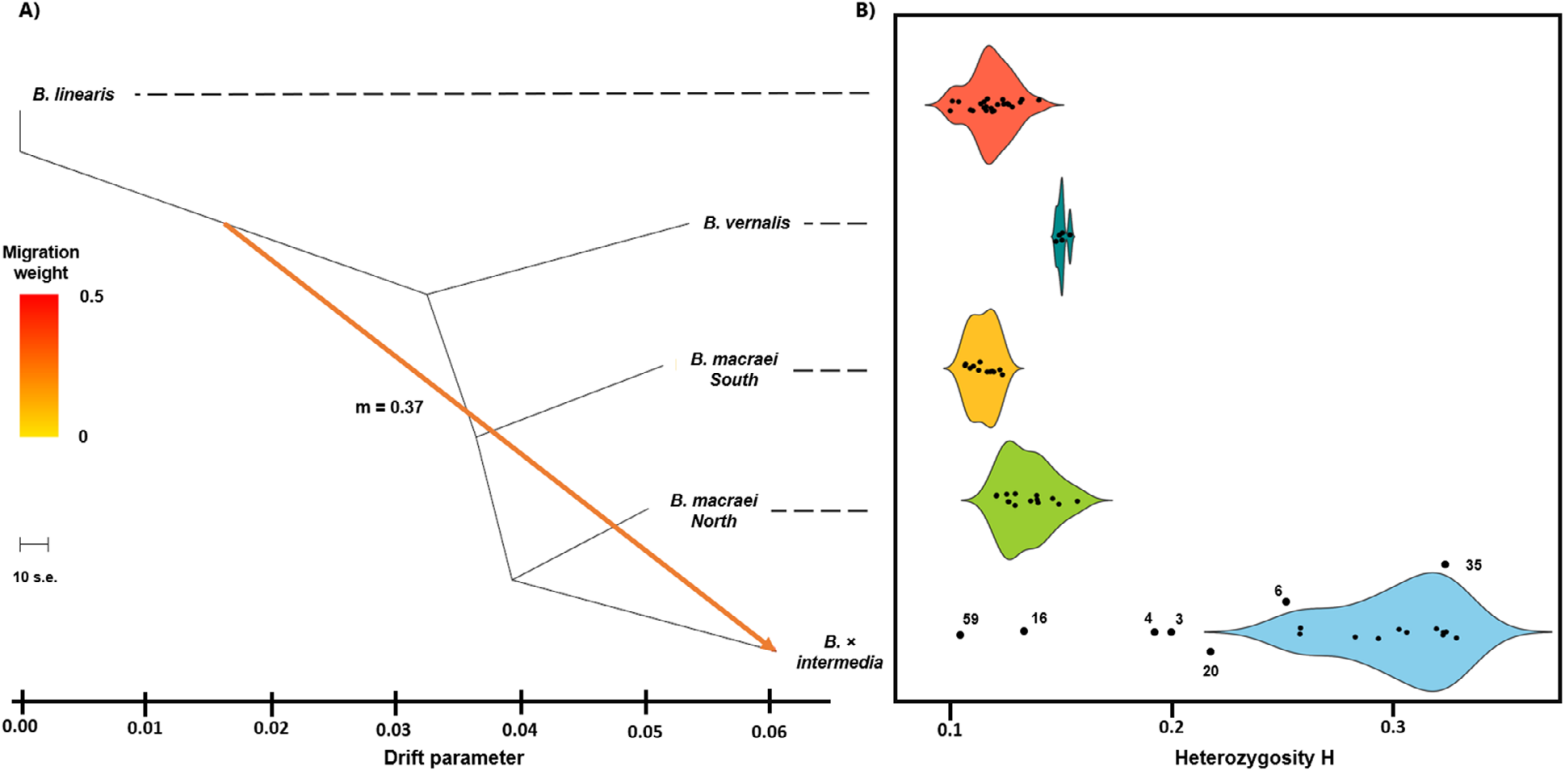
(A) Maximum likelihood tree including one migration event between *Baccharis linearis* and *Baccharis × intermedia* with a migration weight of *m* = 0.37. The arrow is coloured by migration weight, and branch lengths are proportional to genetic drift. The topology indicates a close relationship between *Baccharis macraei*—North and *Baccharis × intermedia*. The analysis was done in “Treemix” v. 1.13. (B) Violin plots of the heterozygosity arranged along the topology of the maximum likelihood tree in (A). *Baccharis × intermedia* has much higher heterozygosity compared to all other taxa. Not assignable individuals are plotted with their individual ID.

In the Treemix analyses, scenarios with different numbers of migration events were tested. In the absence of admixture, the likelihood was found to be lower than in instances where one or more migration events had occurred. Assuming a single migration event increased the likelihood, introducing further migration events, only marginally increased the likelihood (Appendix S5). Therefore, the assumption of one migration event is best supported by the data, considering both parsimony and maximum likelihood. In addition, maximum likelihood residuals were checked for model fit. The population graph (Fig. 4A) shows evidence of admixture between members of *B. linearis* and *B*. × *intermedia*, the latter being closest to the northern *B. macraei* populations. The migration weight (m) had a value of m = 0.37, which indicates the fraction of ancestry from the *B. linearis* donor population. The topology itself was constructed using a maximum likelihood approach. The different groups are well separated. *Baccharis linearis* in this analysis is also separated from the other groups that stand closer to each other than to *B. linearis*. *Baccharis vernalis* is closely related in this topology to *B. macraei*. The values of the drift parameter indicate that *B. macraei* (North and South) and *B. vernalis* originated in close temporal sequence, followed by extended evolution within the different clades.

**Fig. 5:**
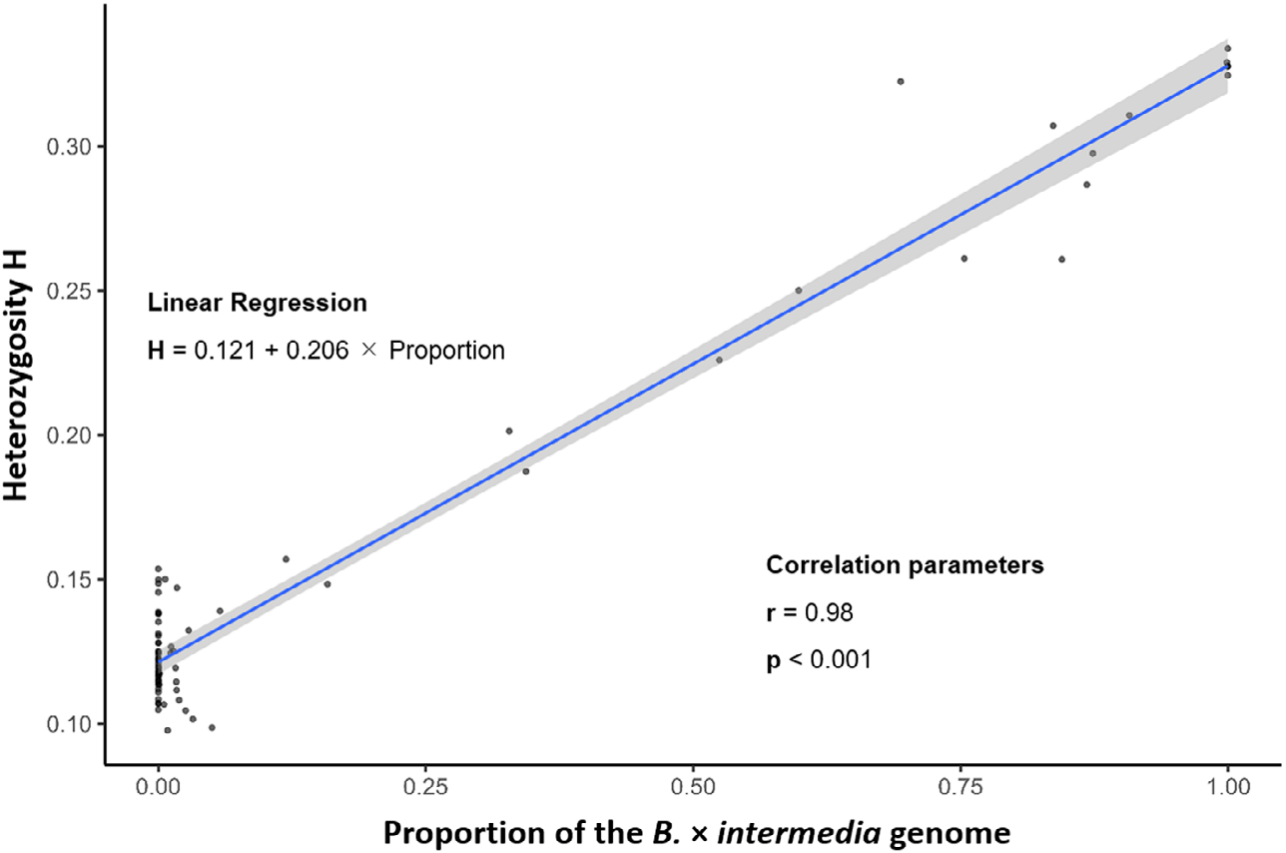
*Baccharis × intermedia* cluster proportion (Fig. 2) and heterozygosity. A generalized linear model (glm) with confidence intervals was used for the regression. Correlation was measured using the “Pearson” method. The two parameters have correlation of *r* = 0.98 and *P* < 0.001.

The heterozygosity values (*H*) of the different groups are shown as a violin plot (Fig. 4B). *Baccharis × intermedia* has by far the highest mean heterozygosity value of nearly *H* = 0.31, ranging from *H* = 0.26 to 0.33. Mean values of the other groups range from *H* = 0.11 to 0.15 (southern *B. macraei* – *B. vernalis*), and the scatter among the data is narrower here than in *B. × intermedia*. From both *B. macraei* groups, the northern group, which is more closely connected to *B. × intermedia*, has a slightly higher heterozygosity mean value (*H* = 0.13) than the southern group (*H* = 0.11). Excluding *B. × intermedia*, *B. vernalis* has the highest heterozygosity of the remaining groups (*H* = 0.19). *Baccharis linearis* has a mean *H* of 0.11. Not assignable individuals were also plotted, and their heterozygosity values are in Fig. 4B. Higher proportions of the “*B. × intermedia* cluster” are strongly correlated (*r* = 0.98) with the amount of heterozygosity (Fig. 5).

Using the program “DSuite”, Patterson's *D*‐statistic was calculated to determine gene flow between different clades in the topologies. Part of *B. linearis* was defined as an outgroup. *D*‐statistic values ≠ 0 and *Z*‐values > |3| indicate introgression (Zheng & Janke 2018).

Each topology is presented as: P1, P2, P3, and O, related by the rooted tree [(P1, P2, P3), O]. *D*‐statistic values >0 (i.e., excess ABBA) indicate gene flow between P2 and P3, and values <0 (i.e., excess BABA) between P1 and P3 (Durand *et al*. 2011; Pease & Hahn 2015). The outgroup O carries the ancestral allele labelled A. The derived allele is labelled B. The patterns are ordered so that the pattern BBAA refers to P1 and P2 sharing the derived allele, ABBA to P2 and P3 sharing the derived allele, and BABA to P1 and P3 sharing the derived allele. Under the null hypothesis, which assumes no gene flow, the ABBA and BABA patterns are expected to be a result of incomplete lineage sorting with equal frequencies (Durand *et al*. 2011; Patterson *et al*. 2012).

The results of this above analysis are presented in Table 4. In the topology [(*B. macraei* South, *B. macraei* North, *B. × intermedia*), Outgroup] gene flow is indicated between *B. macraei* North and *B. × intermedia*, with a *D*‐value of 0.178 and a *z*‐score of 12.53. Also, gene flow was detected between *B. vernalis* (P2) and *B. macraei* North (P3) with *B. × intermedia* as P1, resulting in a *D*‐value of 0.419 and *z*‐score of 31.97. In this case, an *f*_4_ ratio was also calculated: 0.559. Additionally, gene flow is indicated in the topologies [(*B. vernalis*, *B. macraei South*, *B. × intermedia*), Outgroup] with a *D*‐value of 0.368 and *z*‐score of 21.91, and in [(*B. macraei* North, *B. macraei* South, *B. vernalis*), Outgroup] with a respective *D*‐value of 0.055, *z*‐value of 3.268, as well as an *f*_4_ ratio of 0.048. Furthermore, the topologies [(*B. × intermedia*, *B. macraei* North, *B. linearis*), Outgroup] and [(*B. × intermedia*, *B. macraei* South, *B. linearis*), Outgroup] both indicate introgression between P2 and P3, with respective significant *D*‐values of 0.089 and 0.102. In all topologies, the *f*_4_ ratio was not mentioned, as it was zero. All other topologies had a non‐significant *D*‐statistic and a *f*_4_ ratio ≠ 0.

**Table 4:** The *D* statistic and *f*_4_ ratio, with *z*‐score and *P*‐value, as well as BBAA, ABBA, BABA‐values that apply to biallelic SNPs across four populations of *Baccharis* spp. in Chile: P1, P2, P3, and O, related by the rooted tree [(P1, P2, P3), O].

P1	P2	P3	*D* statistic	*Z* score	*P* value	*f*_4_ ratio	BBAA	ABBA	BABA	Introgression detected
*B*. × *intermedia*	*B. macraei* North	*B. linearis*	0.089	7.478	<0.001	0	1615.62	207.58	173.53	X
*B*. × *intermedia*	*B. macraei* South	*B. linearis*	0.102	8.127	<0.001	0	1527.28	218.99	178.47	X
*B*. × *intermedia*	*B. vernalis*	*B. linearis*	0.040	2.998	0.002	0	1326.62	209.16	192.99	
*B. macraei* South	*B. macraei* North	*B*. × *intermedia*	0.178	12.530	0	0	1271.77	278.35	194.40	X
*B*. × *intermedia*	*B. vernalis*	*B. macraei* North	0.419	31.966	0	0.559	157.94	1090.92	446.91	X
*B. vernalis*	*B. macraei* South	*B*. × *intermedia*	0.368	21.910	0	0	1132.16	376.84	174.05	X
*B. macraei* North	*B. macraei* South	*B. linearis*	0.043	2.756	0.006	0	2463.43	77.25	70.85	
*B. vernalis*	*B. macraei* North	*B. linearis*	0.106	<0.001	0	0	2128.50	95.85	77.54	
*B. vernalis*	*B. macraei* South	*B. linearis*	0.138	<0.001	0	0	2141.99	96.89	73.42	
*B. macraei* North	*B. macraei* South	*B. vernalis*	0.055	3.268	0.001	0.048	597.07	291.99	261.61	X

The *f*_3_ ratios for every topology were slightly positive and significant (Appendix S6). There were no signs for admixture among the *f*_3_ values. The only case where the *f*_3_ statistic for a population that is truly admixed fails to be negative is when the population has experienced a high degree of population‐specific genetic drift after the admixture occurred (Patterson *et al*. 2012).

## DISCUSSION

The PCA and clustering show clearly separated groups corresponding to the taxa *Baccharis macraei*, *B. linearis*, *B. vernalis*, and *B. × intermedia*, thus positively answering question (1). *Baccharis macraei* is divided into a northern and southern cluster. This may be explained by genetic differentiation based on geographic distance. The southern and northern populations are separated by approximately 150 km. With complete sampling of all dune sites across the range of distribution of the species and taking into consideration the continuous variation in morphology (Hellwig 1990), a clinal pattern of intraspecific genetic variation would be expected. This expectation appears even more reasonable, given that individual number 16 from La Laguna (i.e., from the southernmost population sampled in the north) shows some introgression between the northern and southern clusters.

*Baccharis × intermedia* falls intermediate between *B. macraei* and *B. linearis* in the PCA. In this analysis, *B. × intermedia* and *B. macraei* are bridged by several individuals (numbers 3, 4, 6, and 20) that cannot clearly be assigned to either of the two clusters. Their position may indicate their origin from crosses between the northern *B. macraei* and *B. × intermedia*. This view is also supported by an increased heterozygosity value for those individuals compared to values in the putative parental species.

The backcrossing takes place primarily in the northern population, only one individual (number 35) seems to be linked genetically to the southern *B. macraei* cluster. This observation probably hints at a polytopic occurrence of hybridization events. Since sampling was much denser in the north, the abundance of interspecific gene flow in the south cannot be estimated from our dataset. We expect that more individuals, like number 35, would be found with an extended sampling in the south of central Chile.

Hybrids between *B. vernalis* and *B. macraei* are described in Hellwig (1990) as *B. × septentrionalis* and considered rare. Additionally, Hellwig (1990 p. 343) wrote in his work on hybrids of *B. vernalis*: “Für […] Bastardbildung mit *B. linearis* subsp. *linearis* gibt es Hinweise, doch ist das Material zu dürftig, um ihn zu beschreiben”, assuming hybrids between *B. linearis* and *B. vernalis*. One individual (number 59) showed proportions of *B. macraei*, *B. linearis*, as well as *B. vernalis*. This individual can be seen as a triple hybrid under the assumption of frequent hybridization among Chilean species of *Baccharis*.

Reducing the cluster number from five to two clusters showed a clear distinction between the groups *B. linearis* on one side, and *B. macraei* and *B. vernalis* on the other side. The putative hybrid *B. × intermedia* belongs in equal parts to the former mentioned clusters, supporting the theory of a hybrid origin.

*Baccharis × intermedia* stands intermediate between *B. linearis* and *B. macraei* not only in the PCA but also in sNMF and hierarchical clustering, *F*_ST_ values and neighbour joining tree. The morphologically intermediate position of *B. × intermedia* was observed previously (Candolle & Candolle 1836; Hellwig 1990; Faini *et al*. 1991) and could be paralleled with genetic data in this study.

To provide genetic evidence for the hybrid nature of *B. × intermedia*, we did a Treemix analysis. The results indicate an unambiguous migration event between *Baccharis linearis* and *B. × intermedia*, which, in this analysis, is part of the *B. macraei* north branch for technical reasons. *Baccharis × intermedia* can be seen as truly admixed, making it difficult to ascribe the individuals either to *B. macraei* or *B. linearis*. The admixed new hybrid lineage should be created by the junction of the two parental branches, but in Treemix it is attached to only one of the parental species, in this case *B. macraei* (north). *Baccharis × intermedia* receives a migration weight of m = 0.37 from *Baccharis linearis*. Thus, *B. × intermedia* might have originated from *B. linearis* and *B. macraei*, with proportions of 37% and 63%, respectively, from the two parental lineages. It is important to mention that Treemix underestimates migration weights when admixture proportions are high (Pickrell & Pritchard 2012). Hence, the migration weight between *B. linearis* and *B. × intermedia* might be even higher than m = 0.37, and may even come close to 0.5.

Because of the position in the PCA, the results from the clustering, and heterozygosity values, it is likely that the *B. × intermedia* cluster itself reflects the composition of the F1 generation. Thus, most of the individuals marked as “unassignable” seem to be backcrosses between *B. macraei* and *B. × intermedia* F1 generation. However, putative backcrosses are not observed between *B. linearis* and the *B. × intermedia* cluster. The observation that individuals ascribed to the *B. × intermedia* cluster are closer to *B. macraei* than to *B. linearis* may be related to the lack of backcrosses between *B. linearis* and the hybrid.

Schumer *et al*. (2014) present different evolutionary scenarios that can produce genome‐wide signatures of hybridization. For *Baccharis × intermedia* two variants can be considered: (A) hybrid swarms – that lack reproductive isolation from parental species, or (B) reproductively isolated lineages – with reproductive isolation between hybrids and parental lineages. However, since backcrossing between the hybrid and *B. macraei* probably occurs (Fig. 3), reproductive isolation is probably not effective here, but could be between the hybrid and *B. linearis*. Therefore, the scenario of hybrid swarms appears to be plausible, but only limited to one parental lineage. Nevertheless, the hybrid only appears in proximity to both the parental species. It is unclear if the lack of backcrosses to *B. linearis* is the result of a sampling bias or if there are mechanisms (e.g., genetic incompatibilities) that inhibits the gene flow. For the moment, as formation of F1 hybrids between *B. macraei* and *B. linearis* occurs quite frequently, it seems rather improbable that genetic incompatibility originates in these hybrids. Whether genomic conflict between plastid and nuclear genomes my play a role here, remains to be tested.

Increased heterozygosity is also an indicator of admixture. Heterozygosity of the admixed population is at least as high as that of the least heterozygous source population. For two parental species and two alleles, the maximum heterozygosity is always *H* = 0.5 (Boca *et al*. 2020) In *B. × intermedia* a mean heterozygosity value of *H* = 0.31 was observed, while in *B. macraei* the respective value is between *H* = 0.11 (South) and *H* = 0.13 (North). In this study, heterozygosity was positively correlated with the proportion of the hybrid (i.e., *B. × intermedia*) genome in the individuals. Including the individuals initially not assigned to either *B. macraei* or *B. × intermedia* (see above), it becomes clear that heterozygosity reflects the amount of genomic material coming from the hybrid in the genomes of backcrosses. Recombination of the different alleles can be observed in the additive effect of chemical compounds in *B. × intermedia* (Faini *et al*. 1991).

From both *Baccharis macraei* groups the northern one, which is more connected to *B. × intermedia*, has a slightly higher heterozygosity value (*H* = 0.13) than the southern group (*H* = 0.11), probably indicating more intense introgression, which can be also hinted by the genetic composition of the individuals 3,4,6 and 20. However, this needs to be further elaborated.

An open question is also whether the hybrid originated from an incomplete split of the parental lineages or after secondary contact of the two parental species. All species belong to subgenus *Baccharis* but supposedly to different sections: *Baccharis linearis* is believed to belong to Sect. Pedicellatae Heering, *B. vernalis* and *B. macraei* both belong to Sect. Cuneifoliae DC. Yet the relationship of sect. Pedicellatae in subgenus *Baccharis* is unclear, and none of the three species were explicitly tested in the phylogenetic study of Heiden *et al*. (2019). Nevertheless, the division into different sections within the subgenus *Baccharis* coincides with the genetic distance between *B. linearis* and the other species. Separation between *B. linearis* and *B. macraei* in the PCA is larger (PC 1) than between *B. vernalis* and *B. macraei* (PC 2). Morphologically, *B. vernalis* and *B. macraei* are also much more similar than *B. linearis* to these two species (Hellwig 1990). This is well supported by all other analyses performed, such as *F*_ST_ values, hierarchical clustering, neighbour joining tree, as well as the Treemix topology.

In order to decide between the two hypotheses, species distribution and its modification via land‐use change is equally important in the discussion. *Baccharis macraei* and *B. vernalis* both occur near the coast. *Baccharis macraei* dwells on sandy soils, often on primary and secondary dunes, while *B. vernalis* occurs on clayey, rocky slopes. In contrast, *B. linearis* is an inland plant and occurs primarily in former sclerophyllous forest areas and eroded areas. Spread by roads is also an important vector for this species (Hellwig 1990). Due to its occurrence, it can be assumed that with increasing human impact on the landscape, the distribution range of *B. linearis* has expanded significantly, starting to overlap with *B. macraei* and *B. vernalis* at some localities at the coast. The distant genetic relationship of *B. linearis* and *B. macraei* makes a secondary contact more plausible than incomplete lineage separation of two parental species, the latter probably being more likely between *B. macraei* and *B. vernalis*.

The results of the *D* statistic indicate, besides gene flow between *B. linearis* and *B. macraei* and *B. × intermedia* and *B. macraei*, which were also revealed in the Treemix investigation, there is gene flow between *B. macraei* and *B. vernalis*. In the latter case a *D* value of 0.055 and a *f*_4_ ratio of 0.048 indicate possible exchange of genetic material via their hybrid, *B. × septentrionalis*.

Treemix topology also suggests rapid evolutionary divergence between *Baccharis vernalis* and *B. macraei*. The drift parameter, which reflects the amount of genetic drift is (with a value of 0.05) minor between the different groups of northern and southern *B. macraei* and *B. vernalis*. This indicates that there might be a rapid split and then a longer anagenetic evolution within the different clades. *B. vernalis* and *B. macraei* are also distinguished, as discussed above, by the difference in habitat. Based on genetic and morphological similarity, it may be assumed that speciation occurred starting with ecotype differentiation within their common ancestor. This hypothesis, however, remains to be tested based on extended sampling.

This study offers genetic evidence that lends support to the hybrid nature of *Baccharis × intermedia*. The genetic differentiation between the two parent species, *B. linearis* and *B. macraei*, supports the hypothesis of a secondary contact event. Further research should focus on this area and address the evolutionary history and phylogeny of all 16 species and their hybrids in Chile. Additionally, the close relationship of *B. macraei* and *B. vernalis* was corroborated. Further research is recommended to shed light on the processes of speciation and specialization regarding soil types and flowering periods between the two species. The scenario of a hybrid swarm is likely, which raises the question of how hybridization affects the characteristics of the parental species. In addition, it is of interest to determine whether the hybrid swarm could be maintained or whether reinforcement or panmixia might occur.

## AUTHOR CONTRIBUTIONS

FS and FH planned the project with the main conceptual ideas. FS worked out almost all of the technical details, and performed the numerical calculations, while FH provided almost all of the material. FS wrote the manuscript, while FH helped with interpreting the results. All authors discussed the results and commented on the manuscript.

## Supporting information


**Appendix S1.** Sparse non‐negative matrix factorization clustering.
**Appendix S2.** PCA.
**Appendix S3.** WC's‐F_ST_.
**Appendix S4.** Hierarchical clustering and neighbour joining tree.
**Appendix S5.** Treemix analysis.
**Appendix S6.** *f*_3_ –Ratio.
**Appendix S7.** LGC library preparation protocol.
**Appendix S8.** Linkage disequilibrium pruning.
