# Supplementary material for: Detecting hybridization in Chilean species of the genus Baccharis L
Source: Plant Biol (Stuttg). 2024 Dec 9;27(2):255–64. doi: 10.1111/plb.13751 (PMC11846629; doi:10.1111/plb.13751)
Supplement: Supplementary file 1 — Appendix S1. Sparse non‐negative matrix factorization clustering. Appendix S2. PCA. Appendix S3. WC's‐FST. Appendix S4. Hierarchical clustering and neighbour joining tree. Appendix S5. Treemix analysis. Appendix S6. f 3 –Ratio. Appendix S7. LGC library preparation protocol. Appendix S8. Linkage disequilibrium pruning. [file PLB-27-255-s001.zip › Appendix_-_Captions_and_tables.docx]

**Supplementary Material**

This document has the captions for supplementary material figures and tables. **The tables are in this document.**

**Appendix 1 – Sparse Non-Negative Matrix Factorization Clustering**

Appendix 1 - Figure 1 – Cross entropy plots of the genetic clusters from four Baccharis species in Chile A) minimal cross entropy for the clusters: K = 0 – 17, with total range as bars, B) Mean cross entropy for the clusters: K = 0 – 20 with standard error as bars.

Appendix 1 - Figure 2 – Genetic cluster assignment of the investigated four Baccharis taxa in Chile using LEA v. 2.6.0 shown as barplot. Two clusters were retained, which are indicated by different colours. The samples are ordered by preliminary field determination. Baccharis macraei and B. vernalis forming a distinct group, while B. linearis is forming the other group. B. × intermedia clearly showing an intermediate composition of the two clusters.

**Appendix 2 – PCA**

Appendix 2 Table 1 – Principal components, standard deviation and proportion of variance performed with the SNP’s from Baccharis.

| **Principal Component** | **Standard**  **deviation** | **Proportion of Variance** | **Cumulative Proportion** |
| --- | --- | --- | --- |
| PC 1 | 40.48 | 0.414 | 0.414 |
| PC 2 | 17.38 | 0.765 | 0.491 |
| PC 3 | 15.25 | 0.058 | 0.550 |
| PC 4 | 9.19 | 0.021 | 0.572 |
| PC 5 | 8.17 | 0.017 | 0.588 |
| PC 6 | 7.93 | 0.016 | 0.604 |
| PC 7 | 7.30 | 0.013 | 0.618 |
| PC 8 | 7.13 | 0.013 | 0.631 |
| PC 9 | 6.74 | 0.012 | 0.642 |
| PC 10 | 6.63 | 0.011 | 0.653 |

Appendix 2 - Figure 1 – PCA results. Putatively assigned individuals of Baccharis due to field determination into the three species and the hybrid; a) PC 1 (41.1%) and PC 2 (7.6%); b) PC 1 41.1%) and PC 3 (5.8%); c) PC 2 (7.6%) and PC 3 (5.8%).

Appendix 2 - Figure 2 - PCA results-Assigned Baccharis individuals due to the sNMF using K = 5 clusters. Baccharis macraei was split into a northern and southern cluster. Grouping into “Not assignable” was done to individuals that belong more than 25 % to a different group in the sNMF-clustering. a) PC 1 (41.1%) and PC 2 (7.6%); b) PC 1 41.1%) and PC 3 (5.8%); c) PC 2 (7.6%) and PC 3 (5.8%).

**Appendix 3 – WC’s-F_ST_**

Appendix 3 - Table 1 – Genetic distances (WC’s-FST) between species of Baccharis in Chile.

|  | *B. × intermedia* | *B.*  *linearis* | *B. macraei- North* | *B. macraei- South* | *B.*  *vernalis* |
| --- | --- | --- | --- | --- | --- |
| *B. × intermedia* | - |  |  |  |  |
| *B. linearis* | 0.092 | - |  |  |  |
| *B. macraei - North* | 0.067 | 0.136 | - |  |  |
| *B. macraei - South* | 0.089 | 0.146 | 0.039 | - |  |
| *B. vernalis* | 0.114 | 0.155 | 0.084 | 0.092 | - |

**Appendix 4 – Hierarchical Clustering and Neighbour Joining Tree**

Appendix 4 - Figure 1 – Hierarchical Clustering Dendrogram of four Baccharis species in Chile. The “hclust” command was used to create this tree. As a criterion for clustering “Ward.D2” was chosen, representing Ward’s (1963) clustering (Murtagh and Legendre 2014).

Appendix 4 - Figure 2 – Unrooted Neighbor-Joining Tree of four Baccharis species in Chile.

**Appendix 5 – Treemix analysis**

Appendix 5 - Figure 1 – Maximum likelihood trees including different number of migration events (edges) between taxa of Baccharis. The arrow is coloured by migration weight, and branch lengths are proportional to genetic drift. The analysis was done in “Treemix” v. 1.13 and the graph was plotted using the residuals in.

Appendix 5 - Figure 2 – Residual fit between the different groups of Baccharis four species in Chile. Plotted is the residual fit from the maximum likelihood topologies with different migration events (edges) in the next figures. Colours are described in the palette on the right. Residuals above zero represent populations that are more closely related to each other in the data than in the best-fit tree, and thus are candidates for admixture events. The analysis was done in “Treemix” v. 1.13 and the graph was plotted using the residuals in “R” (Pickrell and Pritchard 2012, R Core Team 2019).

Appendix 5 - Figure 3 – ln(likelihood) in comparison of the number of migration events resulting from the “Treemix” analysis.

**Appendix 6 – f_3_ -Ratio**

Table 1 – Estimating f3-ratios for all possible topologies of four Baccharis species in Chile diving the dataset in 7 blocks of size of 500 bp. f3 statistics were calculated with Treemix v. 1.13.

| **Topology** | **f_3_ - ratio** | **standard error** | **z-score** |
| --- | --- | --- | --- |
| B. x intermedia;  B. vernalis, B. linearis | 0.0126802 | 0.000996905 | 12.7195 |
| B. linearis;  B. x intermedia, B. vernalis | 0.0209197 | 0.000936556 | 22.3368 |
| B. vernalis;  B. x intermedia, B. linearis | 0.0327779 | 0.00122447 | 26.7691 |
| B. x intermedia;  B. vernalis, B. macraei South | 0.0230359 | 0.00119168 | 19.3305 |
| B. macraei South;  B. x intermedia, B. vernalis | 0.0162474 | 0.000591617 | 27.4627 |
| B. vernalis;  B. x intermedia, B. macraei South | 0.0224222 | 0.00123332 | 18.1803 |
| B. x intermedia;  B. vernalis, B. macraei North | 0.0213797 | 0.00106706 | 20.0361 |
| B. macraei North;  B. x intermedia, B. vernalis | 0.0139346 | 0.000748856 | 18.6078 |
| B. vernalis;  B. x intermedia, B. macraei North | 0.0240784 | 0.000967652 | 24.8833 |
| B. x intermedia;  B. linearis, B. macraei South | 0.0114555 | 0.00090181 | 12.7028 |
| B. linearis;  B. x intermedia, B. macraei South | 0.0221443 | 0.000515622 | 42.9468 |
| B. macraei South;  B. x intermedia, B. linearis | 0.0278278 | 0.000914476 | 30.4303 |
| B. x intermedia;  B. linearis, B. macraei North | 0.0100273 | 0.000646637 | 15.5069 |
| B. linearis;  B. x intermedia, B. macraei North | 0.0235725 | 0.000700547 | 33.6487 |
| B. macraei North;  B. x intermedia, B. linearis | 0.0252869 | 0.00161134 | 15.6931 |
| B. x intermedia;  B. macraei South, B. macraei North | 0.0229226 | 0.00068994 | 33.224 |
| B. macraei North;  B. x intermedia, B. macraei South | 0.0123916 | 0.00113028 | 10.9633 |
| B. macraei South;  B. x intermedia, B. macraei North | 0.0163607 | 0.000581226 | 28.1485 |
| B. linearis;  B. vernalis, B. macraei South | 0.0325 | 0.00108952 | 29.8298 |
| B. macraei South;  B. vernalis, B. linearis | 0.0174721 | 0.00108244 | 16.1414 |
| B. vernalis;  B. linearis, B. macraei South | 0.0211975 | 0.00061071 | 34.7097 |
| B. linearis;  B. vernalis, B. macraei North | 0.032272 | 0.00118315 | 27.2764 |
| B. macraei North;  B. vernalis, B. linearis | 0.0165874 | 0.001209 | 13.72 |
| B. vernalis;  B. linearis, B. macraei North | 0.0214256 | 0.000706141 | 30.3418 |
| B. macraei North;  B. vernalis, B. macraei South | 0.0140478 | 0.000806109 | 17.4267 |
| B. macraei South;  B. vernalis, B. macraei North | 0.0147044 | 0.000515889 | 28.5031 |
| B. vernalis;  B. macraei South, B. macraei North | 0.0239652 | 0.000935912 | 25.6062 |
| B. linearis;  B. macraei South, B. macraei North | 0.0350396 | 0.000916469 | 38.2333 |
| B. macraei North;  B. linearis, B. macraei South | 0.0138198 | 0.00108991 | 12.6797 |
| B. macraei South;  B. linearis B. macraei North | 0.0149325 | 0.000882681 | 16.9172 |

**Appendix 7 – LGC Library preparation protocol**

*Baccharis* (Asteraceae) using Pst-ApeKI

**I. Restriction digest:**

20-500 ng of genomic DNA were digested with 2 Units each ApekI and PstI-HF(NEB) in 1 times NEBuffer 3.1 in 20μl volume for 30 min at 37°C. The restriction enzymes were heat inactivated by incubation at 75°C for 30 min.

**II. nGBS library construction:**

**a) Ligation Reaction**

10 μl of each restriction digest were transferred to a new 96-well PCR plate, mixed on ice first with 1.5 μl of one of 96 inline-barcoded forward PstI Adaptors (pre-hybridized, concentration 1 pM/μl), followed by addition of 20μl Ligation master mix (contains: 15 μl NEB Quick ligation buffer, 0.4 μl NEB Quick Ligase, 5 pM pre-hybridized common reverse ApekI Adaptors). Ligation reactions were incubated for 1h at RT, followed by heat inactivation for 10 min at 65°C.

**b) Library purification**

all reactions were diluted with 30 μl TE 10/50 (10mM Tris/HCl, 50mM EDTA, pH:8.0) and mixed with 50 μl Agencourt XP beads, incubated for 10 min at RT and placed for 5 min on a magnet to collect the beads. The supernatant was discarded, and the beads were washed two times with 200 μl 80% Ethanol. Beads were air dried for 10 min and libraries were eluted in 15 μl Tris Buffer (10 mM Tris/HCl pH:9)

**c) Library amplification**

10 μl of each of the 96 Libraries were separately amplified in 20 μl PCR reactions using MyTaq (Bioline) and standard Illumina TrueSeq amplification primers. Cycle number was limited to 16 Cycles.

**III. Pooling and clean up of ddRAD libraries:**

5 μl from each of the 96 amplified libraries were pooled. PCR primer and small amplicons were removed by Agencourt XP bead purification using 0.8 Volume of beads. The PCR enzyme was removed by an additional purification on Qiagen MinElute Columns. The pooled library was eluted in a final volume of 20μl Tris Buffer (5 mM Tris/HCl pH:9).

**IV. Normalisation**

Normalisation was done using Trimmer Kit (Evrogen). 1 μg pooled GBS library in 12 μl was mixed with 4 μl 4x hybridization buffer, denatured for 3 min at 98°C and incubated for 3 hours at 68°C to allow reassociation of DNA fragments. 20 μl of 2x DSN master buffer was added and the samples were incubated for 10 min at 66°C. One Unit of DSN enzyme (1U/μl) was added and the reaction was incubated for another 40 min. Reaction was terminated by the addition of 20μl DSN Stop Solution, purified on a Qiagen MinElute Column and eluted in 10μl Tris Buffer (5 mM Tris/HCl pH:9).

**V. Reamplification**

The normalized library pool was reamplified in 100μl PCR reactions using MyTaq (Bioline). An i5-Adaptor primer was used to include an i5-Index into the library, allowing parallel sequencing of multiple libraries on the Illumina NextSeq 500/550 sequencer. Cycle number was limited to 14 cycles.

**Appendix 8 – Linkage Disequilibrium pruning**

**Linkage Disequilibrium**

Linkage disequilibrium (LD) is a population-based parameter that describes the degree to which two alleles are linked and inherited together within a given population. The pruning of highly linked alleles has been demonstrated to improve the correlation and quality of subsequent analysis (Abdellaoui et al., 2013). The R package GWLD (version 1.3.4) was used to test LD, with R (version 4.0.3) being the computational environment (Zhang et al., 2023). For each main species (population in a broader sense) involved, the presence of linkage disequilibrium (LD) was investigated. SNPs with a higher reduced mutual information (RMI) value of >0.7 were removed. In *Baccharis linearis*, 520 SNPs were linked. In *B. macraei*, 715 were linked, while no SNPs were linked in *B. vernalis*. A total of 1150 unique SNPs were removed from the dataset, leaving 9865 SNPs for further analysis.

**Results**

PCA, Clustering, Heterozygosity and Nei-F_ST_ (see App. 8 – Tables and Graphs) was done anew and has shown only very minor changes, mostly in the second and third decimal place. Therefore, the core message of the paper does not change and the

**References**

Abdellaoui, A., Hottenga, JJ., Knijff, P. *et al.* (2013) Population structure, migration, and diversifying selection in the Netherlands. *European Journal of Human Genetics* **21**, 1277–1285, https://doi.org/10.1038/ejhg.2013.48

Zhang, R., Wu, H., Li, Y., Huang, Z., Yin, Z., Yang, C. X., & Du, Z. Q. (2023). GWLD: an R package for genome-wide linkage disequilibrium analysis. *G3 (Bethesda, Md.)*, **13**(9), jkad154. <https://doi.org/10.1093/g3journal/jkad154>

Appendix 8 - Figure 1 – Cross entropy plots of the genetic clusters from four Baccharis species in Chile Mean cross entropy for the clusters: K = 0 – 20 with standard error as bars.

Appendix 8 - Figure 2 – Genetic cluster assignment of the investigated four Baccharis taxa in Chile. Two clusters were retained, which are indicated by different colours. The samples are ordered by preliminary field determination. Baccharis macraei and B. vernalis forming a distinct group, while B. linearis is forming the other group. B. × intermedia clearly showing an intermediate composition of the two clusters.

Appendix 8 - Figure 3 – PCA results. Assigned Baccharis individuals due to the sNMF using K = 5 clusters. Baccharis macraei was split into a northern and southern cluster. Grouping into “Not assignable” was done to individuals that belong more than 25 % to a different group in the sNMF-clustering. PC 1 (43.2%) and PC 2 (7.7%).

Appendix 8 - Figure 4 – The violin plots of the heterozygosity. *Baccharis × intermedia* has a much higher heterozygosity value compared to all the other taxa. Not assignable individuals are plotted with their number representing the individual ID.

Appendix 8 - Table 1 – Genetic distances (Nei-FST) between species of Baccharis in Chile.

|  | *B. × intermedia* | *B.*  *linearis* | *B. macraei- North* | *B. macraei- South* | *B.*  *vernalis* |
| --- | --- | --- | --- | --- | --- |
| *B. × intermedia* | - |  |  |  |  |
| *B. linearis* | 0.091 | - |  |  |  |
| *B. macraei - North* | 0.068 | 0.136 | - |  |  |
| *B. macraei - South* | 0.089 | 0.145 | 0.034 | - |  |
| *B. vernalis* | 0.112 | 0.157 | 0.085 | 0.092 | - |
