# Supplementary figures and images for: Detecting hybridization in Chilean species of the genus Baccharis L
Source: Plant Biol (Stuttg). 2024 Dec 9;27(2):255–64. doi: 10.1111/plb.13751 (PMC11846629; doi:10.1111/plb.13751)

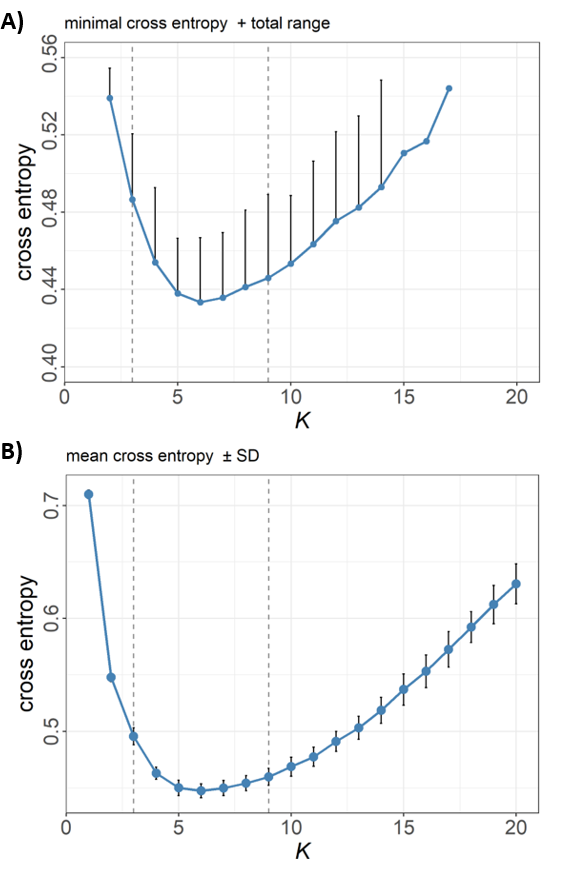

Supplement: Supplementary file 1 — Appendix S1. Sparse non‐negative matrix factorization clustering. Appendix S2. PCA. Appendix S3. WC's‐FST. Appendix S4. Hierarchical clustering and neighbour joining tree. Appendix S5. Treemix analysis. Appendix S6. f 3 –Ratio. Appendix S7. LGC library preparation protocol. Appendix S8. Linkage disequilibrium pruning. [file PLB-27-255-s001.zip › Appendix 1-_Figure_1.tif]

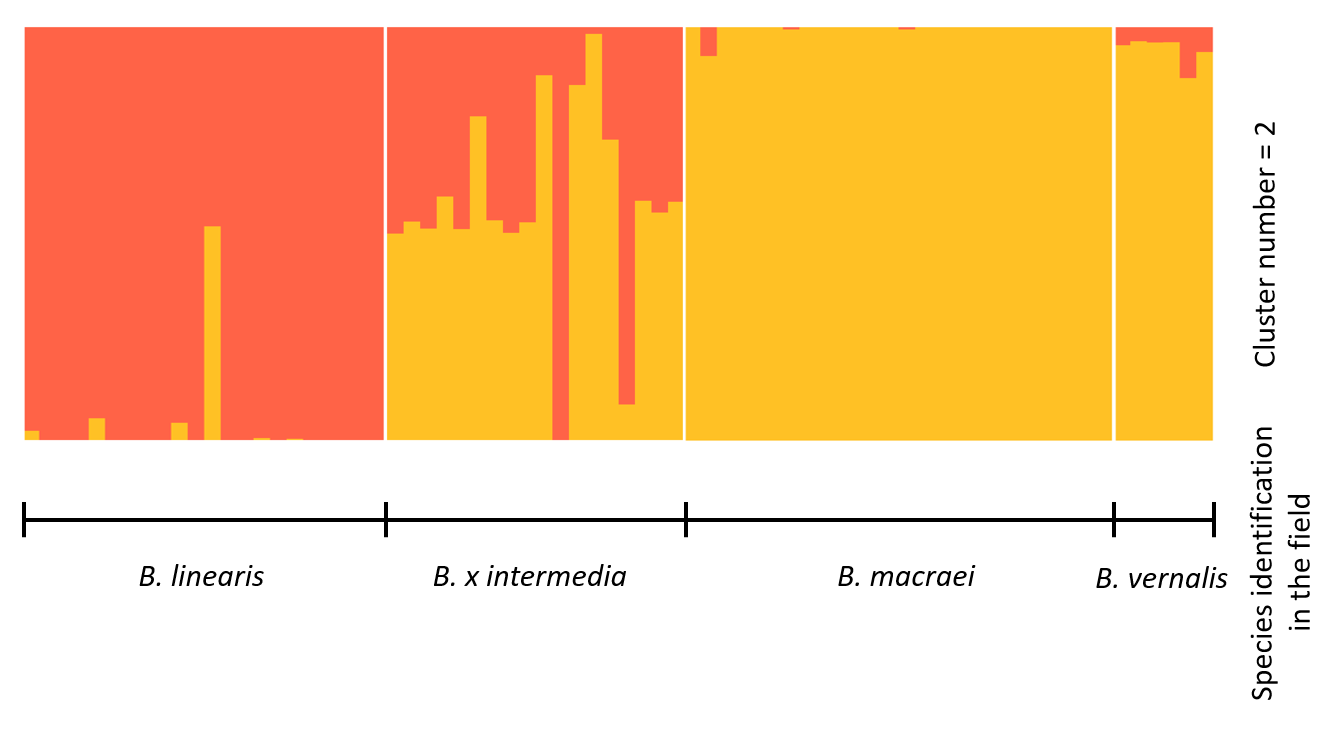

Supplement: Supplementary file 1 — Appendix S1. Sparse non‐negative matrix factorization clustering. Appendix S2. PCA. Appendix S3. WC's‐FST. Appendix S4. Hierarchical clustering and neighbour joining tree. Appendix S5. Treemix analysis. Appendix S6. f 3 –Ratio. Appendix S7. LGC library preparation protocol. Appendix S8. Linkage disequilibrium pruning. [file PLB-27-255-s001.zip › Appendix 1-_Figure_2.tif]

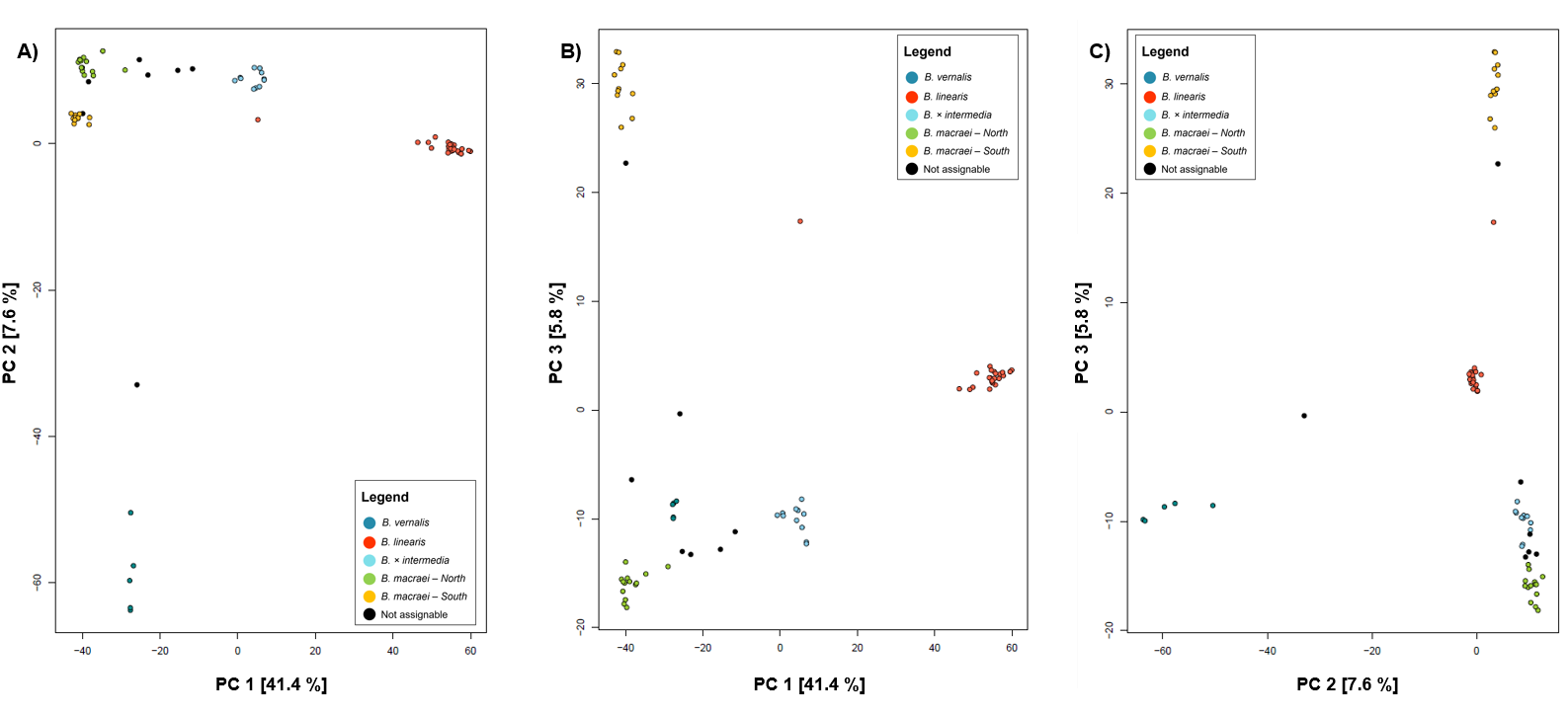

Supplement: Supplementary file 1 — Appendix S1. Sparse non‐negative matrix factorization clustering. Appendix S2. PCA. Appendix S3. WC's‐FST. Appendix S4. Hierarchical clustering and neighbour joining tree. Appendix S5. Treemix analysis. Appendix S6. f 3 –Ratio. Appendix S7. LGC library preparation protocol. Appendix S8. Linkage disequilibrium pruning. [file PLB-27-255-s001.zip › Appendix 2- Figure 2.tif]

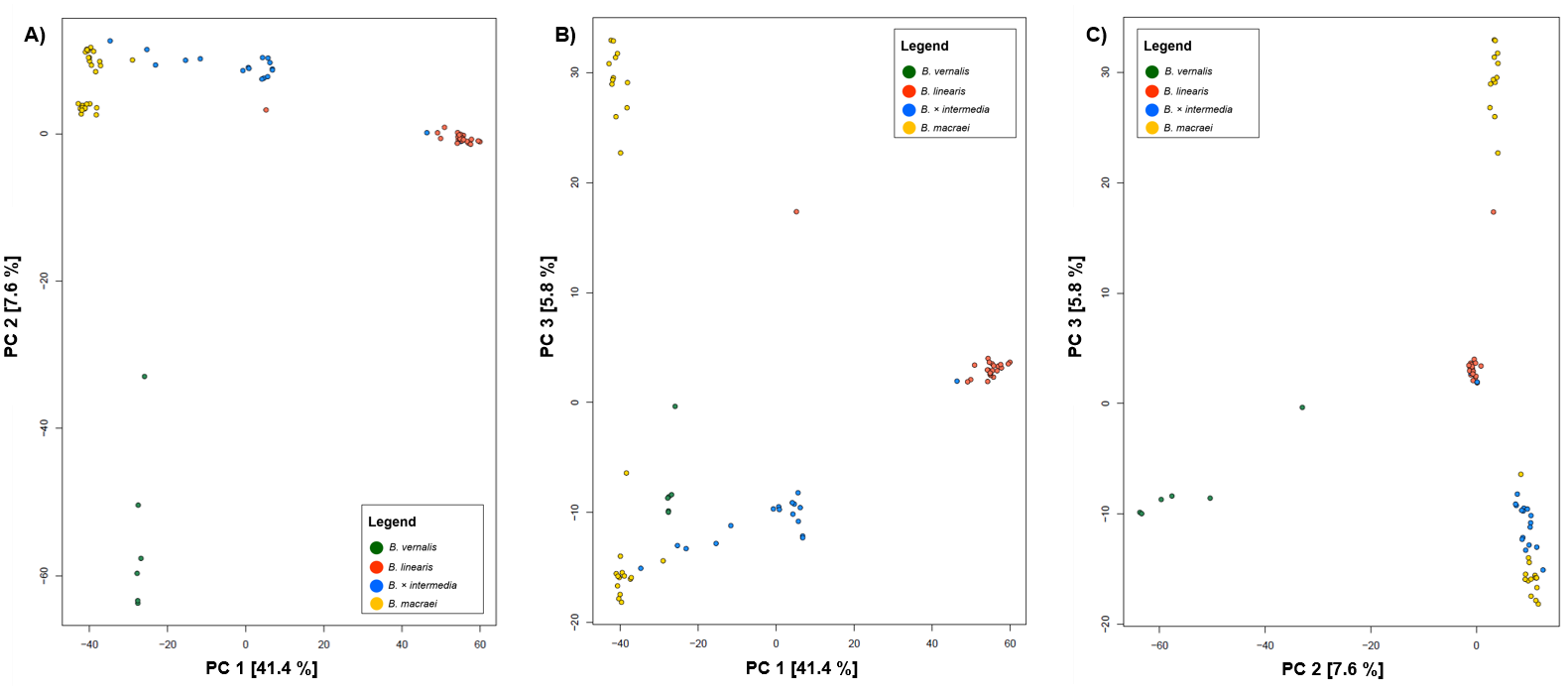

Supplement: Supplementary file 1 — Appendix S1. Sparse non‐negative matrix factorization clustering. Appendix S2. PCA. Appendix S3. WC's‐FST. Appendix S4. Hierarchical clustering and neighbour joining tree. Appendix S5. Treemix analysis. Appendix S6. f 3 –Ratio. Appendix S7. LGC library preparation protocol. Appendix S8. Linkage disequilibrium pruning. [file PLB-27-255-s001.zip › Appendix 2-Figure_1.tif]

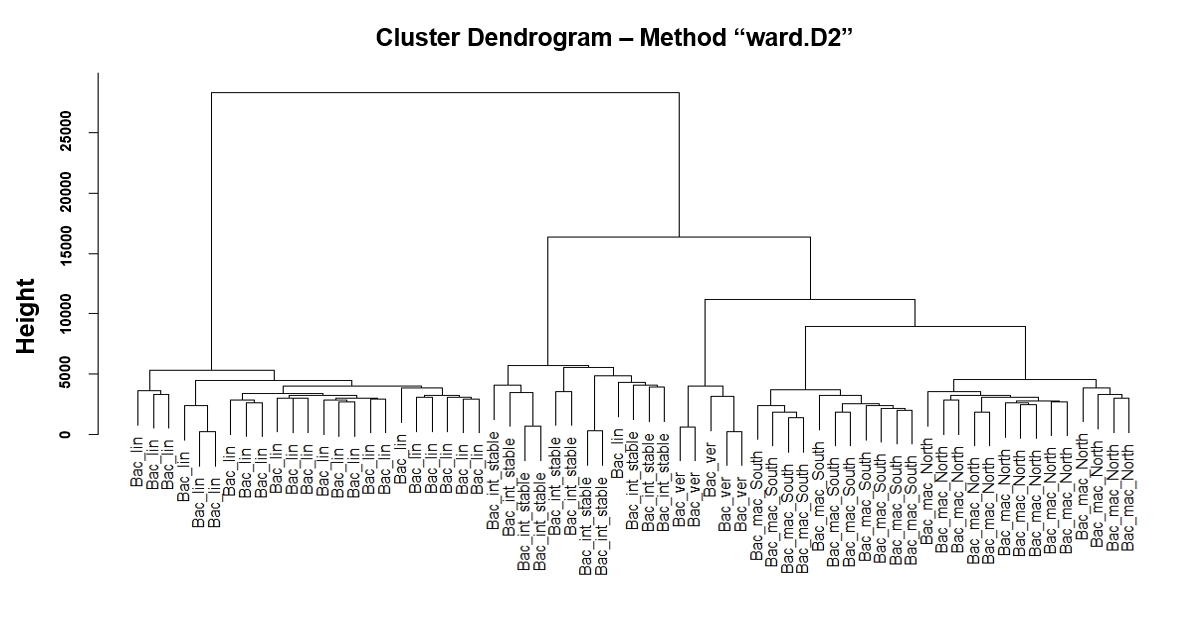

Supplement: Supplementary file 1 — Appendix S1. Sparse non‐negative matrix factorization clustering. Appendix S2. PCA. Appendix S3. WC's‐FST. Appendix S4. Hierarchical clustering and neighbour joining tree. Appendix S5. Treemix analysis. Appendix S6. f 3 –Ratio. Appendix S7. LGC library preparation protocol. Appendix S8. Linkage disequilibrium pruning. [file PLB-27-255-s001.zip › Appendix 4- Figure 1.tif]

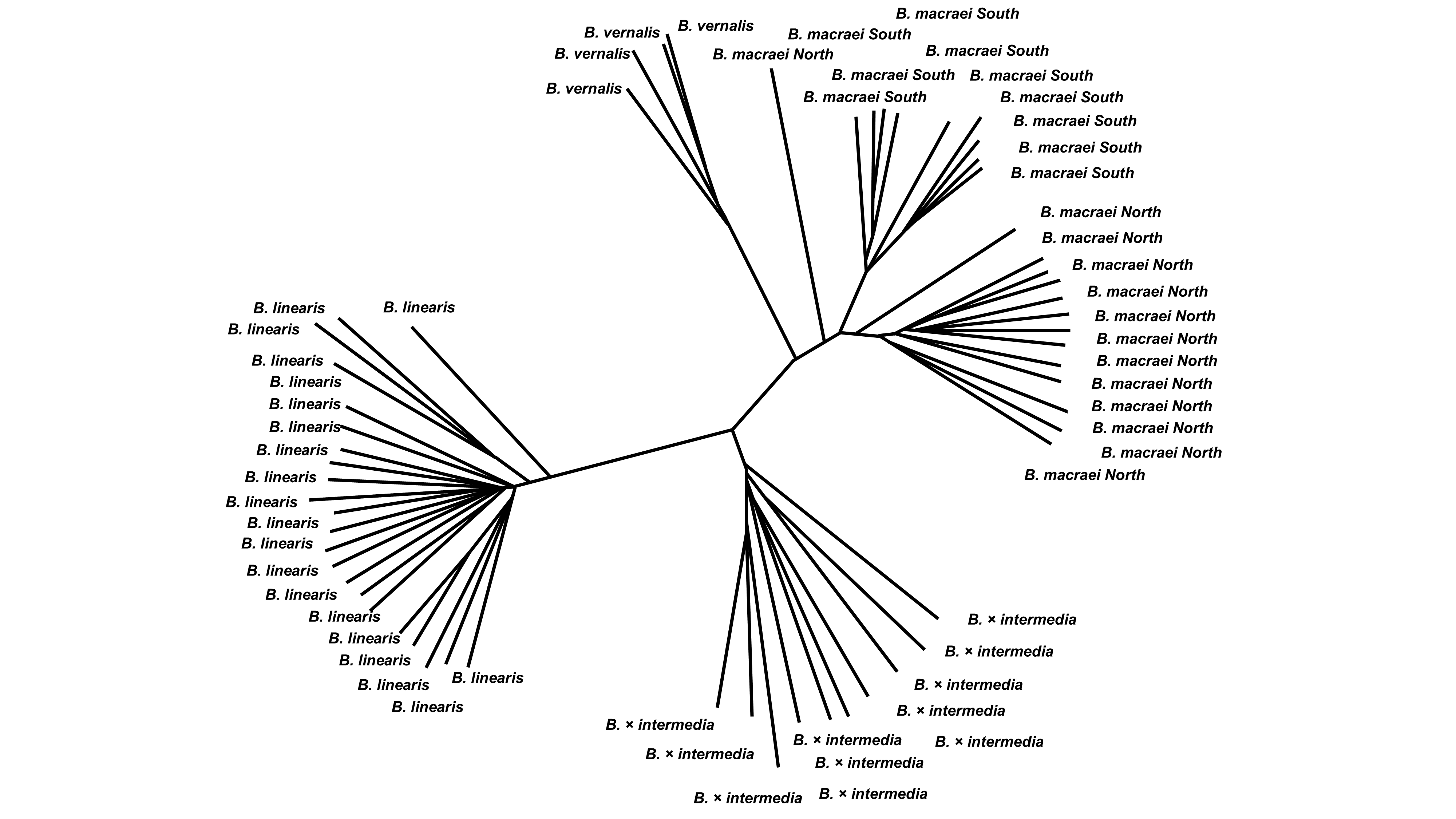

Supplement: Supplementary file 1 — Appendix S1. Sparse non‐negative matrix factorization clustering. Appendix S2. PCA. Appendix S3. WC's‐FST. Appendix S4. Hierarchical clustering and neighbour joining tree. Appendix S5. Treemix analysis. Appendix S6. f 3 –Ratio. Appendix S7. LGC library preparation protocol. Appendix S8. Linkage disequilibrium pruning. [file PLB-27-255-s001.zip › Appendix 4- Figure 2.tif]

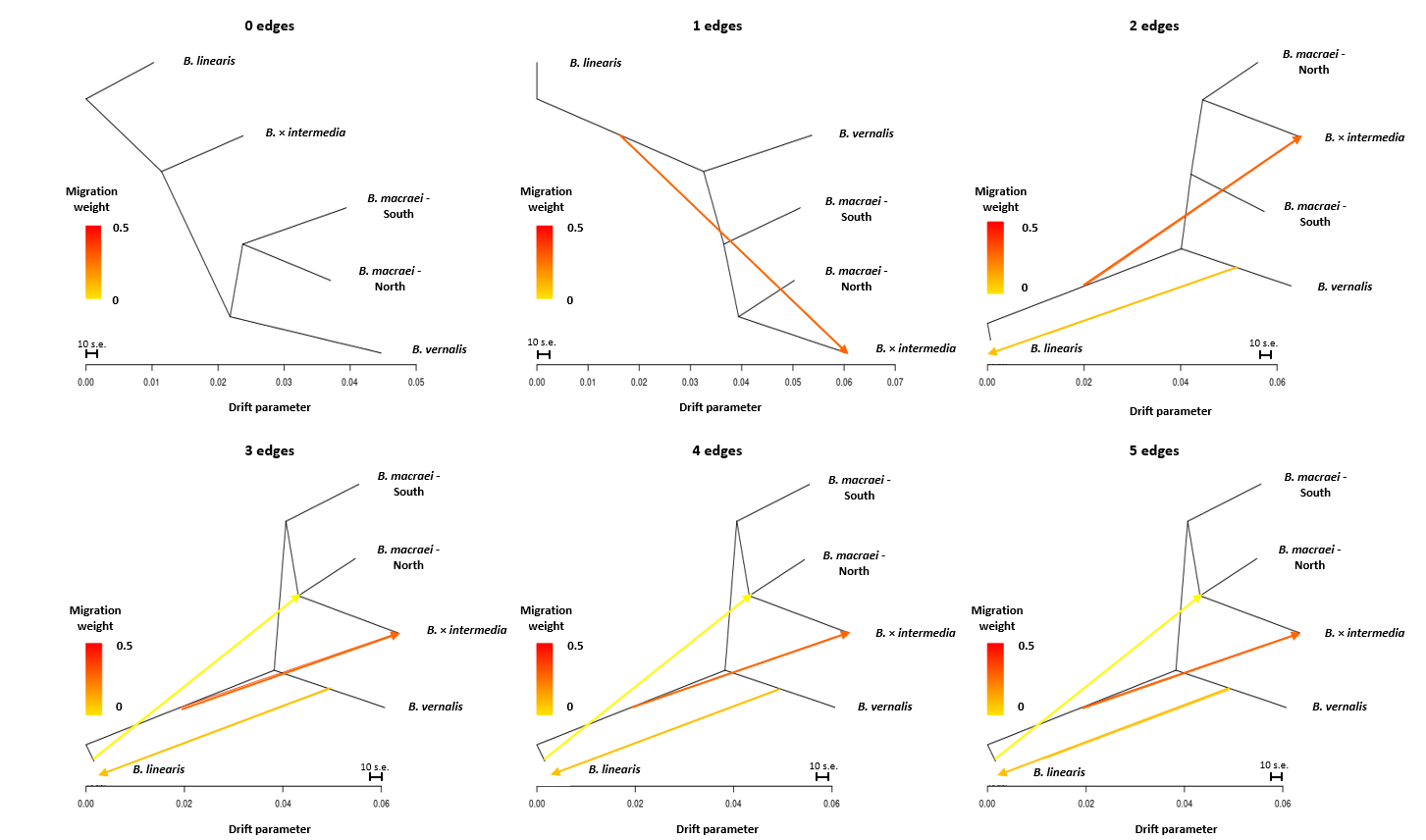

Supplement: Supplementary file 1 — Appendix S1. Sparse non‐negative matrix factorization clustering. Appendix S2. PCA. Appendix S3. WC's‐FST. Appendix S4. Hierarchical clustering and neighbour joining tree. Appendix S5. Treemix analysis. Appendix S6. f 3 –Ratio. Appendix S7. LGC library preparation protocol. Appendix S8. Linkage disequilibrium pruning. [file PLB-27-255-s001.zip › Appendix 5 - Figure 1.tif]

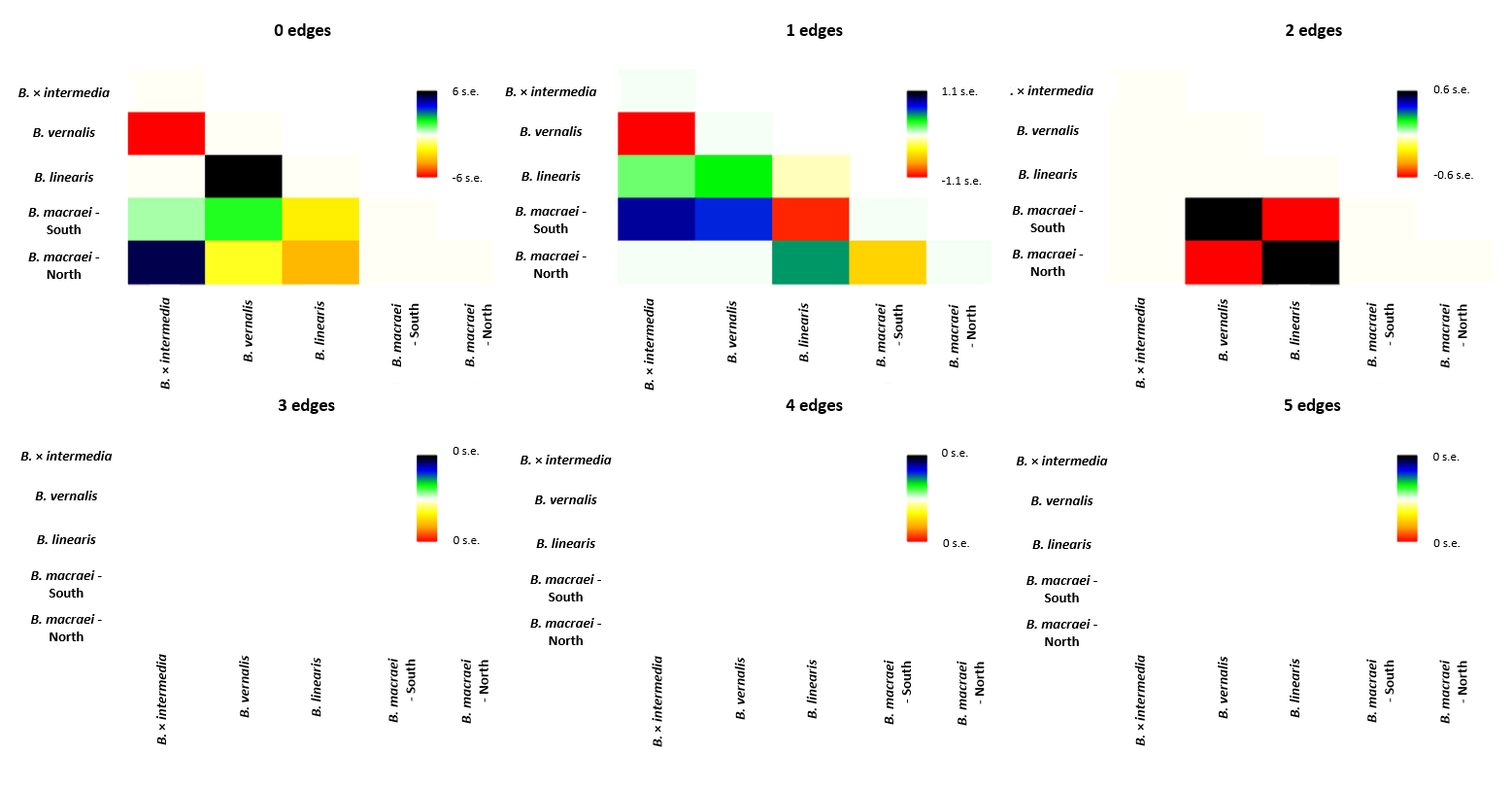

Supplement: Supplementary file 1 — Appendix S1. Sparse non‐negative matrix factorization clustering. Appendix S2. PCA. Appendix S3. WC's‐FST. Appendix S4. Hierarchical clustering and neighbour joining tree. Appendix S5. Treemix analysis. Appendix S6. f 3 –Ratio. Appendix S7. LGC library preparation protocol. Appendix S8. Linkage disequilibrium pruning. [file PLB-27-255-s001.zip › Appendix 5 - Figure 2.tif]

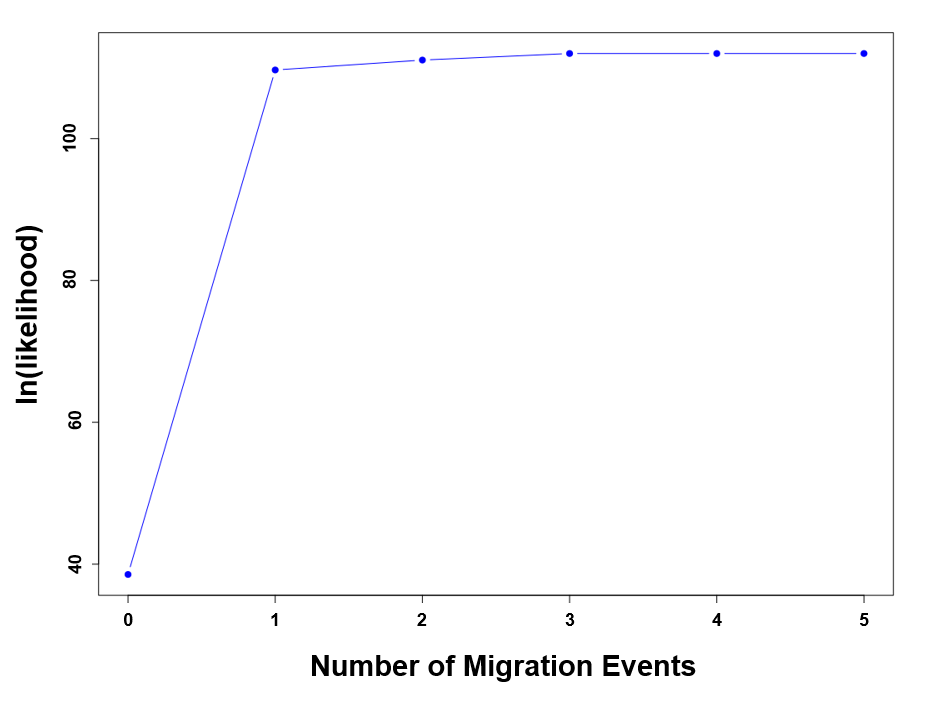

Supplement: Supplementary file 1 — Appendix S1. Sparse non‐negative matrix factorization clustering. Appendix S2. PCA. Appendix S3. WC's‐FST. Appendix S4. Hierarchical clustering and neighbour joining tree. Appendix S5. Treemix analysis. Appendix S6. f 3 –Ratio. Appendix S7. LGC library preparation protocol. Appendix S8. Linkage disequilibrium pruning. [file PLB-27-255-s001.zip › Appendix 5 - Figure 3.tif]

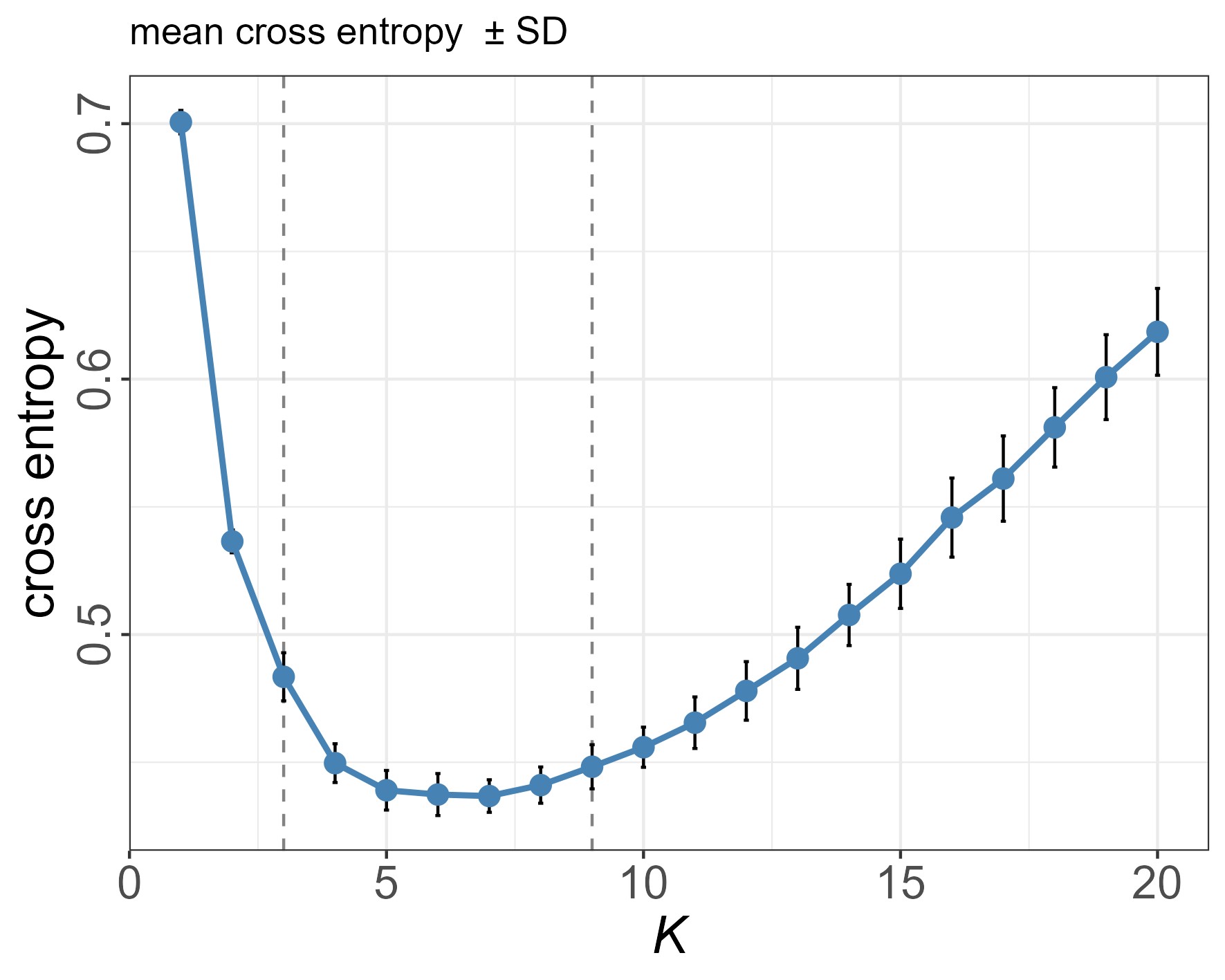

Supplement: Supplementary file 1 — Appendix S1. Sparse non‐negative matrix factorization clustering. Appendix S2. PCA. Appendix S3. WC's‐FST. Appendix S4. Hierarchical clustering and neighbour joining tree. Appendix S5. Treemix analysis. Appendix S6. f 3 –Ratio. Appendix S7. LGC library preparation protocol. Appendix S8. Linkage disequilibrium pruning. [file PLB-27-255-s001.zip › Appendix 8 - Figure 1.jpg]

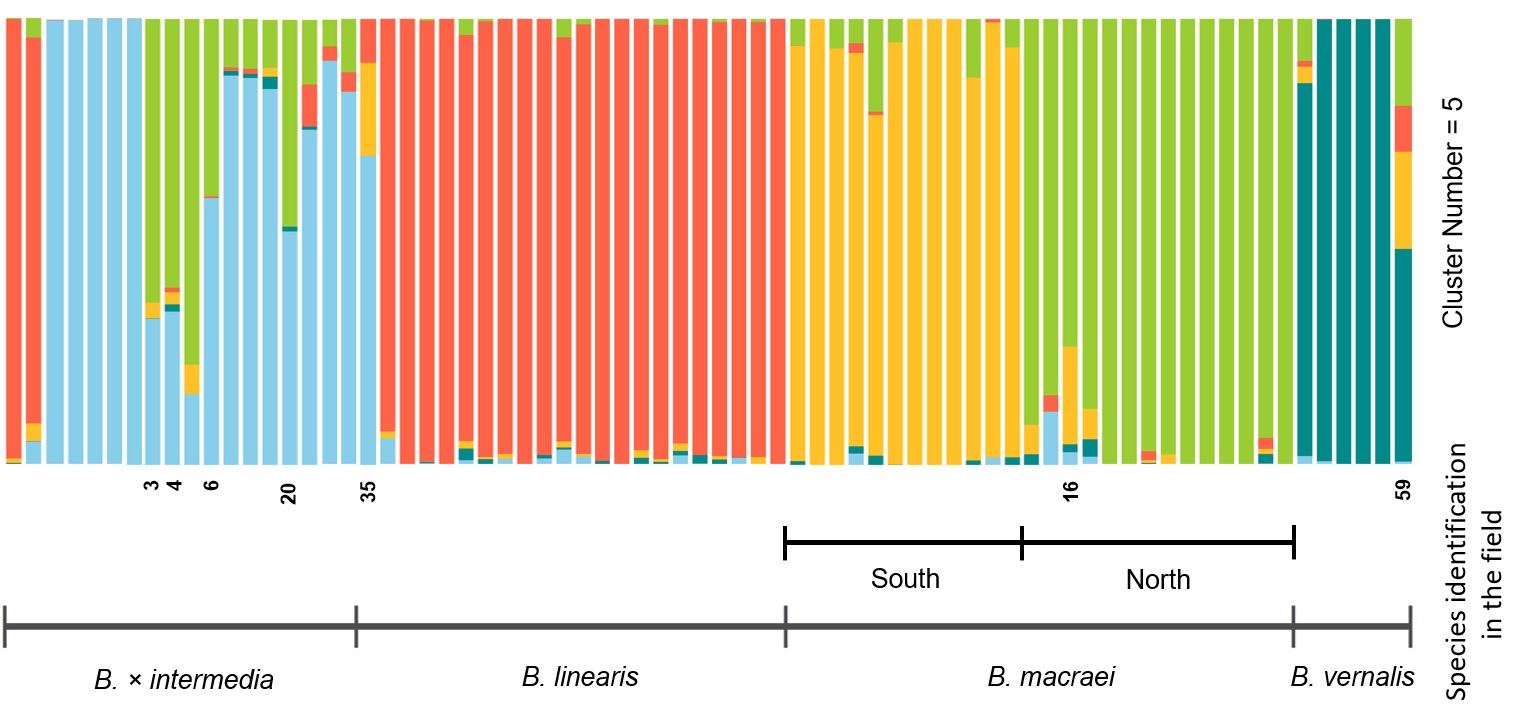

Supplement: Supplementary file 1 — Appendix S1. Sparse non‐negative matrix factorization clustering. Appendix S2. PCA. Appendix S3. WC's‐FST. Appendix S4. Hierarchical clustering and neighbour joining tree. Appendix S5. Treemix analysis. Appendix S6. f 3 –Ratio. Appendix S7. LGC library preparation protocol. Appendix S8. Linkage disequilibrium pruning. [file PLB-27-255-s001.zip › Appendix 8 - Figure 2.jpg]

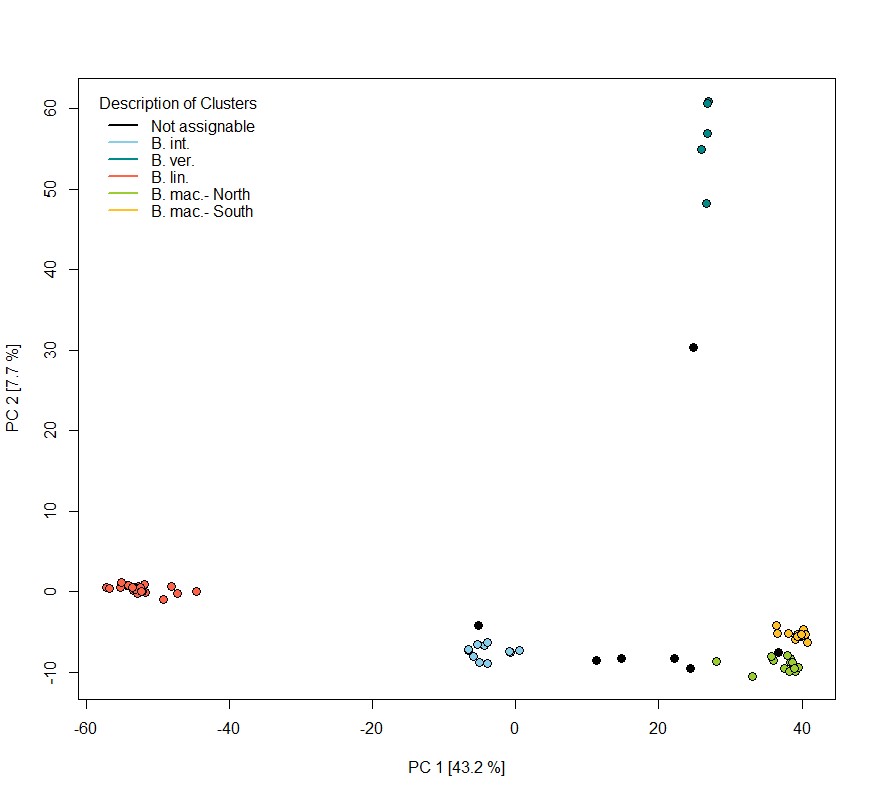

Supplement: Supplementary file 1 — Appendix S1. Sparse non‐negative matrix factorization clustering. Appendix S2. PCA. Appendix S3. WC's‐FST. Appendix S4. Hierarchical clustering and neighbour joining tree. Appendix S5. Treemix analysis. Appendix S6. f 3 –Ratio. Appendix S7. LGC library preparation protocol. Appendix S8. Linkage disequilibrium pruning. [file PLB-27-255-s001.zip › Appendix 8 - Figure 3.jpg]

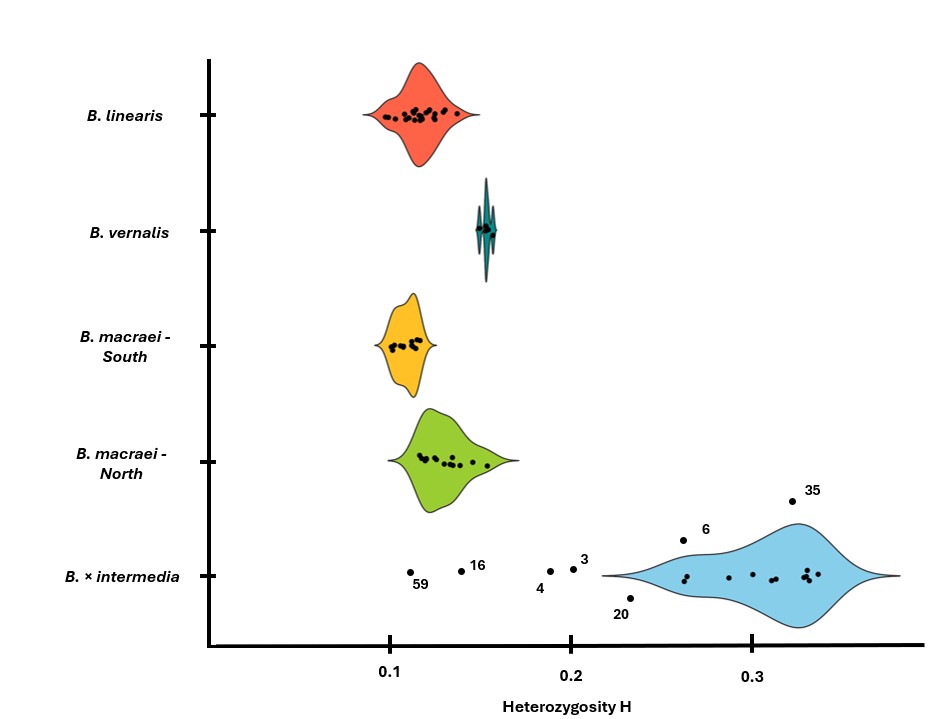

Supplement: Supplementary file 1 — Appendix S1. Sparse non‐negative matrix factorization clustering. Appendix S2. PCA. Appendix S3. WC's‐FST. Appendix S4. Hierarchical clustering and neighbour joining tree. Appendix S5. Treemix analysis. Appendix S6. f 3 –Ratio. Appendix S7. LGC library preparation protocol. Appendix S8. Linkage disequilibrium pruning. [file PLB-27-255-s001.zip › Appendix 8 - Figure 4.jpg]
